# Supplementary material for: Enhanced Tomato Yellow Leaf Curl Thailand Virus Suppression Through Multi-Disease and Insect-Resistant Tomato Lines Combining Virus and Vector Resistance
Source: Insects. 2025 Jul 15;16(7):721. doi: 10.3390/insects16070721 (PMC12295848; doi:10.3390/insects16070721)
Supplement: Supplementary file 1 [file insects-16-00721-s001.zip › File S2. Raw gel electrophoresis images.pdf]

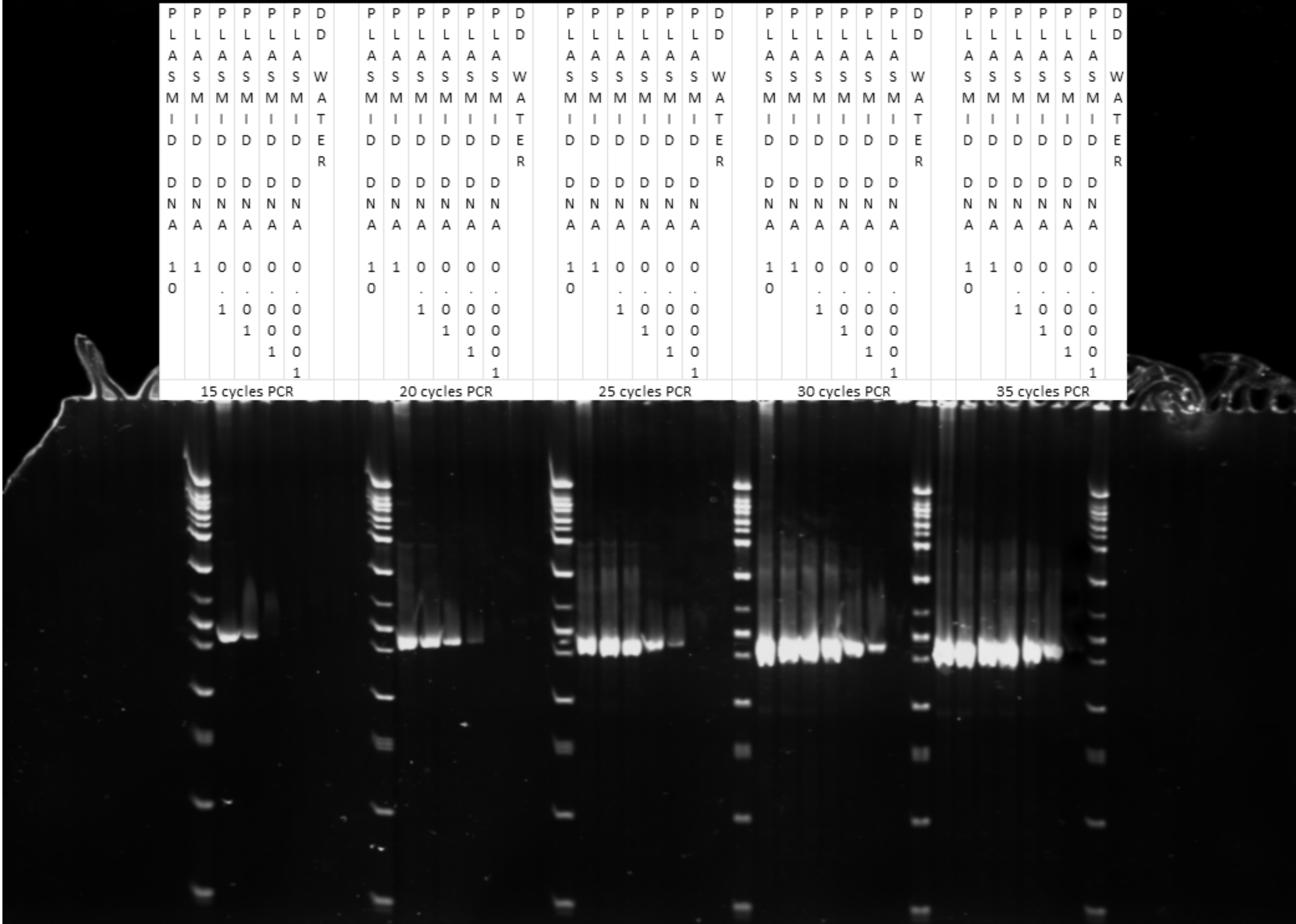
PLASMID DNA 10
PLASMID DNA 1
PLASMID DNA 0.1
PLASMID DNA 0.01
PLASMID DNA 0.001
PLASMID DNA 0.0001
DD WATER
15 cycles PCR
PLASMID DNA 10
PLASMID DNA 1
PLASMID DNA 0.1
PLASMID DNA 0.01
PLASMID DNA 0.001
PLASMID DNA 0.0001
DD WATER
20 cycles PCR
PLASMID DNA 10
PLASMID DNA 1
PLASMID DNA 0.1
PLASMID DNA 0.01
PLASMID DNA 0.001
PLASMID DNA 0.0001
DD WATER
25 cycles PCR
PLASMID DNA 10
PLASMID DNA 1
PLASMID DNA 0.1
PLASMID DNA 0.01
PLASMID DNA 0.001
PLASMID DNA 0.0001
DD WATER
30 cycles PCR
PLASMID DNA 10
PLASMID DNA 1
PLASMID DNA 0.1
PLASMID DNA 0.01
PLASMID DNA 0.001
PLASMID DNA 0.0001
DD WATER
35 cycles PCR

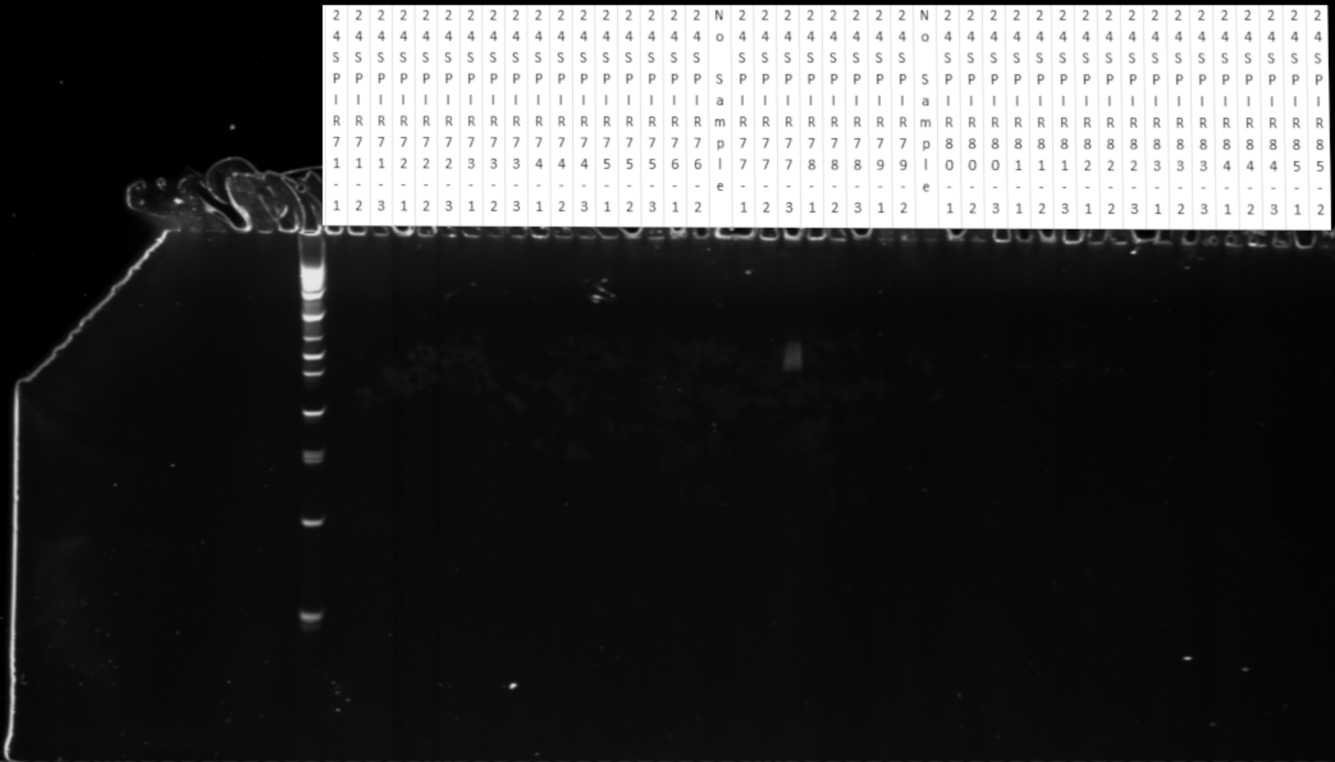
24SPIR71-1
24SPIR71-2
24SPIR71-3
24SPIR72-1
24SPIR72-2
24SPIR72-3
24SPIR73-1
24SPIR73-2
24SPIR73-3
24SPIR74-1
24SPIR74-2
24SPIR74-3
24SPIR75-1
24SPIR75-2
24SPIR75-3
24SPIR76-1
24SPIR76-2
No Sample
24SPIR77-1
24SPIR77-2
24SPIR77-3
24SPIR78-1
24SPIR78-2
24SPIR78-3
24SPIR79-1
24SPIR79-2
No Sample
24SPIR80-1
24SPIR80-2
24SPIR80-3
24SPIR81-1
24SPIR81-2
24SPIR81-3
24SPIR82-1
24SPIR82-2
24SPIR82-3
24SPIR83-1
24SPIR83-2
24SPIR83-3
24SPIR84-1
24SPIR84-2
24SPIR84-3
24SPIR85-1
24SPIR85-2

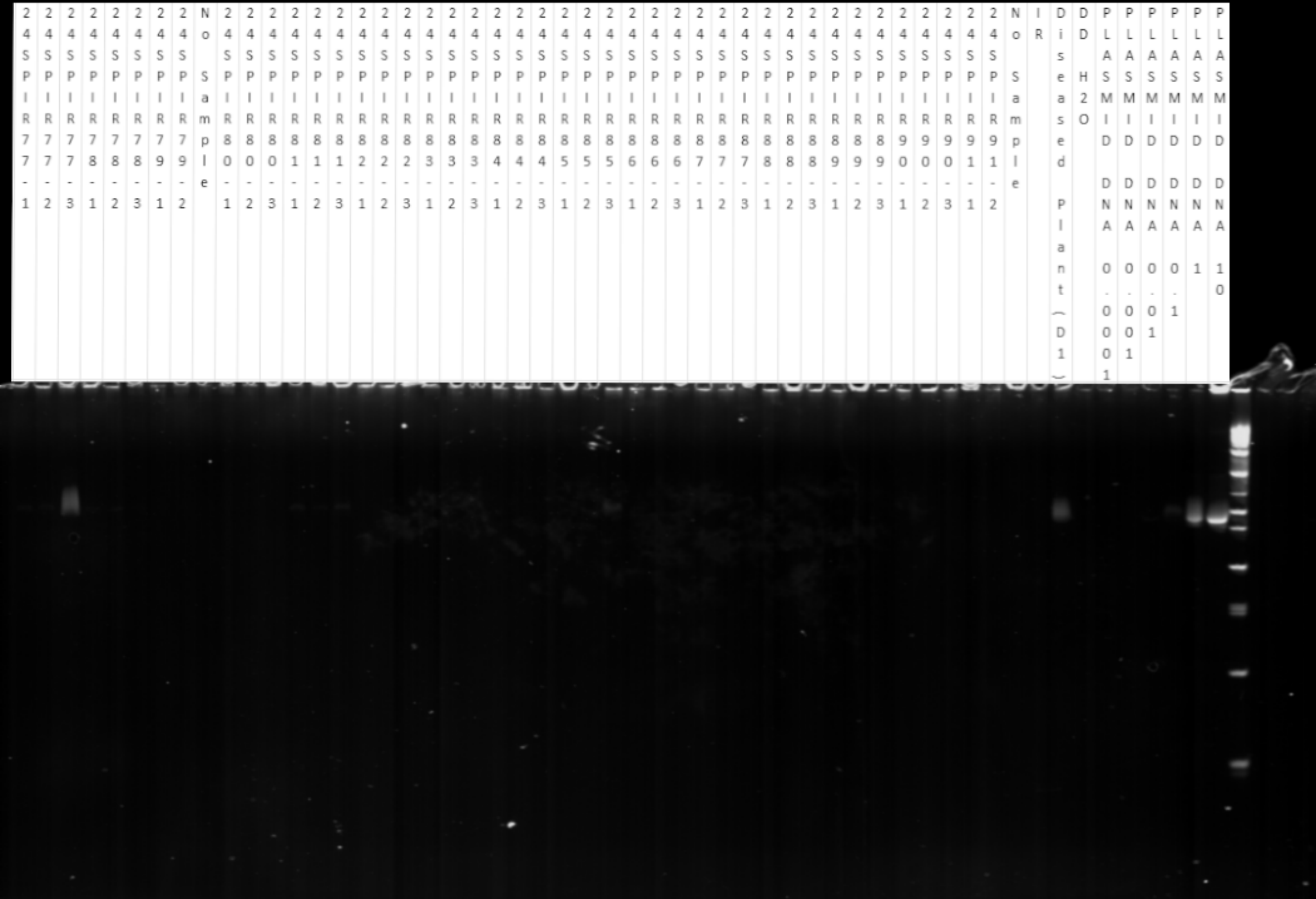
24SPIR77-1
24SPIR77-2
24SPIR77-3
24SPIR78-1
24SPIR78-2
24SPIR78-3
24SPIR79-1
24SPIR79-2
No Sample
24SPIR80-1
24SPIR80-2
24SPIR80-3
24SPIR81-1
24SPIR81-2
24SPIR81-3
24SPIR82-1
24SPIR82-2
24SPIR82-3
24SPIR83-1
24SPIR83-2
24SPIR83-3
24SPIR84-1
24SPIR84-2
24SPIR84-3
24SPIR85-1
24SPIR85-2
24SPIR85-3
24SPIR86-1
24SPIR86-2
24SPIR86-3
24SPIR87-1
24SPIR87-2
24SPIR87-3
24SPIR88-1
24SPIR88-2
24SPIR88-3
24SPIR89-1
24SPIR89-2
24SPIR89-3
24SPIR90-1
24SPIR90-2
24SPIR90-3
24SPIR91-1
24SPIR91-2
No Sample
IR
Diseased Plant (D1)
DD H2O
PLASMID DNA 0.0001
PLASMID DNA 0.001
PLASMID DNA 0.01
PLASMID DNA 0.1
PLASMID DNA 1
PLASMID DNA 10

20240510
PCR program: SSR60-20 cycles
Electrophoresis: 6% acrylamide 50min
Day 3 PCR

24SPIR71-1	24SPIR71-2	24SPIR71-3	24SPIR72-1	24SPIR72-2	24SPIR72-3	24SPIR73-1	24SPIR73-2	24SPIR73-3	24SPIR74-1	24SPIR74-2	24SPIR74-3	24SPIR75-1	24SPIR75-2	24SPIR75-3	24SPIR76-1	24SPIR76-2	No Sample	24SPIR77-1	24SPIR77-2	24SPIR77-3	24SPIR78-1	24SPIR78-2	24SPIR78-3	24SPIR79-1	24SPIR79-2	No Sample	24SPIR80-1	24SPIR80-2	24SPIR80-3	24SPIR81-1	24SPIR81-2	24SPIR81-3	24SPIR82-1	24SPIR82-2	24SPIR82-3	24SPIR83-1	24SPIR83-2	24SPIR83-3	24SPIR84-1	24SPIR84-2	24SPIR84-3	24SPIR85-1	24SPIR85-2	24SPIR85-3	24SPIR86-1	24SPIR86-2	24SPIR86-3	24SPIR87-1	24SPIR87-2

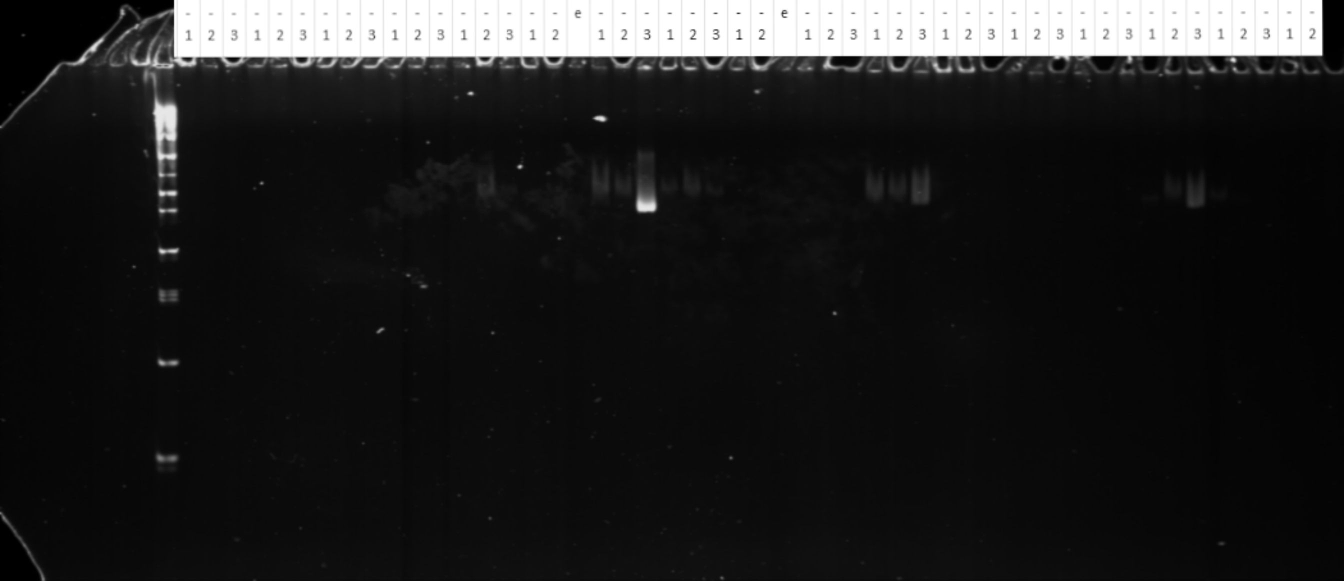

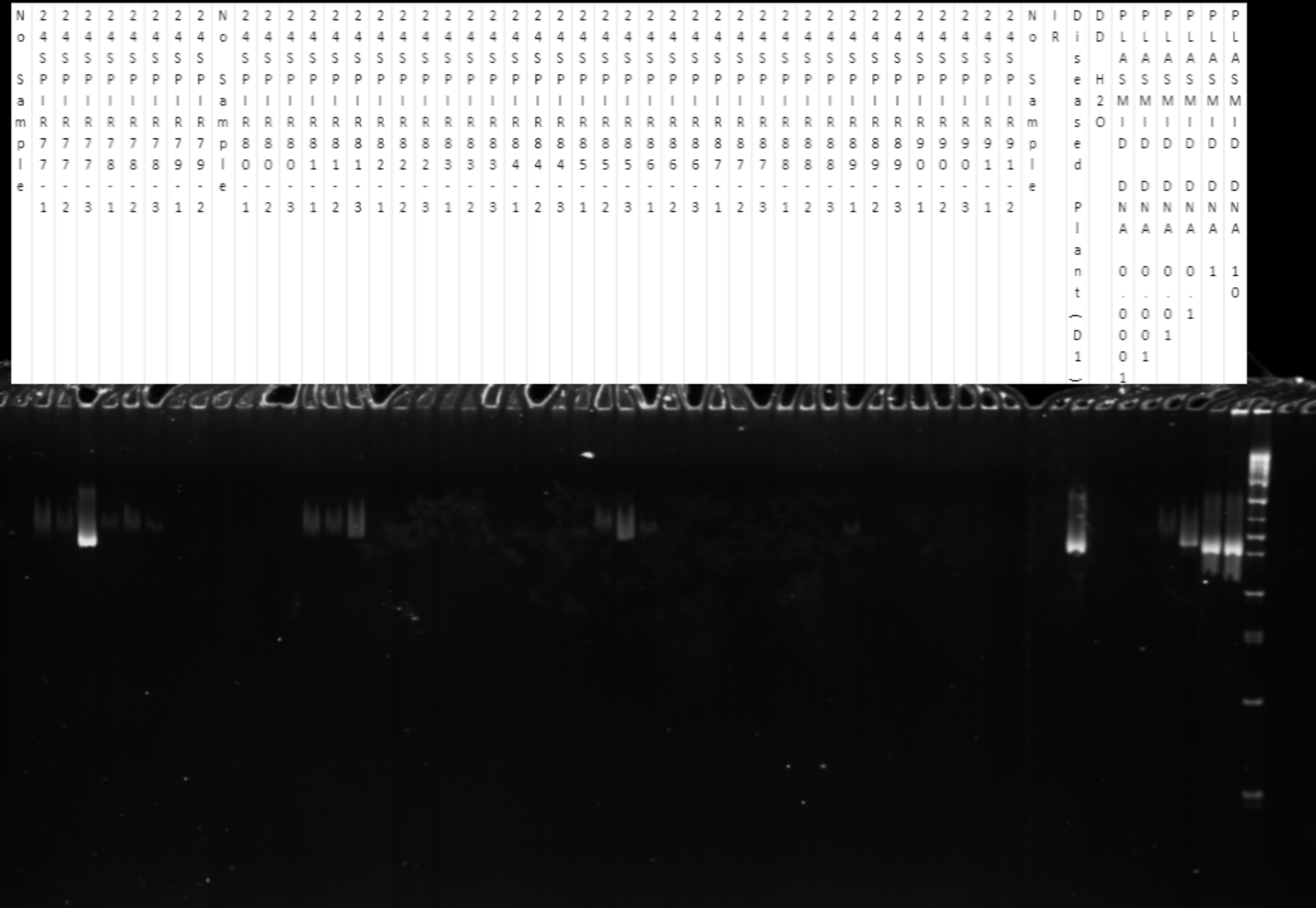
No Sample
24SPIR77-1
24SPIR77-2
24SPIR77-3
24SPIR78-1
24SPIR78-2
24SPIR78-3
24SPIR79-1
24SPIR79-2
No Sample
24SPIR80-1
24SPIR80-2
24SPIR80-3
24SPIR81-1
24SPIR81-2
24SPIR81-3
24SPIR82-1
24SPIR82-2
24SPIR82-3
24SPIR83-1
24SPIR83-2
24SPIR83-3
24SPIR84-1
24SPIR84-2
24SPIR84-3
24SPIR85-1
24SPIR85-2
24SPIR85-3
24SPIR86-1
24SPIR86-2
24SPIR86-3
24SPIR87-1
24SPIR87-2
24SPIR87-3
24SPIR88-1
24SPIR88-2
24SPIR88-3
24SPIR89-1
24SPIR89-2
24SPIR89-3
24SPIR90-1
24SPIR90-2
24SPIR90-3
24SPIR91-1
24SPIR91-2
No Sample
IR
Diseased Plant (D1)
DD H2O
PLASMID DNA 0.0001
PLASMID DNA 0.001
PLASMID DNA 0.01
PLASMID DNA 0.1
PLASMID DNA 1
PLASMID DNA 10

PCR program: SSR60-25 cycles	
Electrophoresis: 6% acrylamide 50min	
Day 3 PCR	

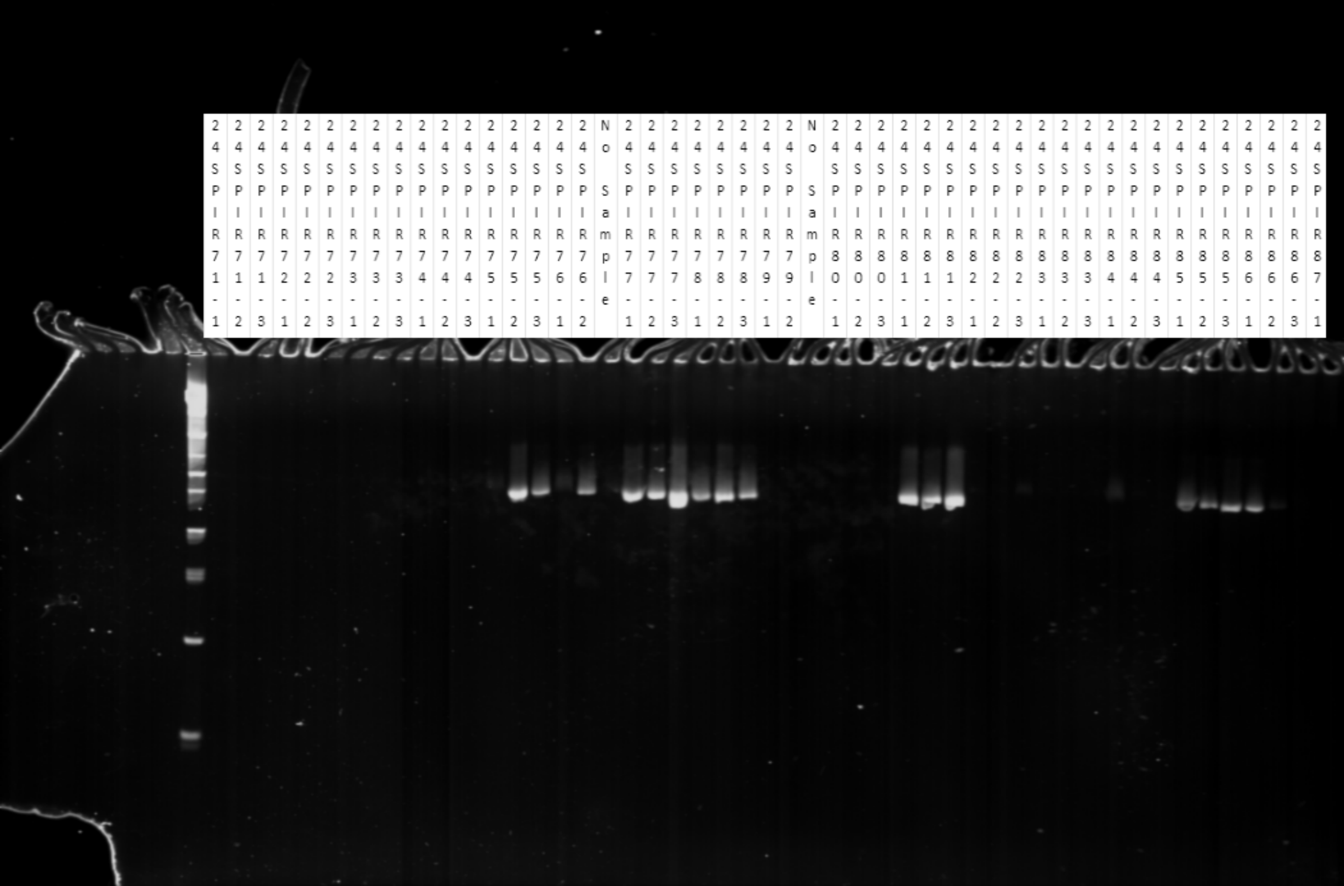

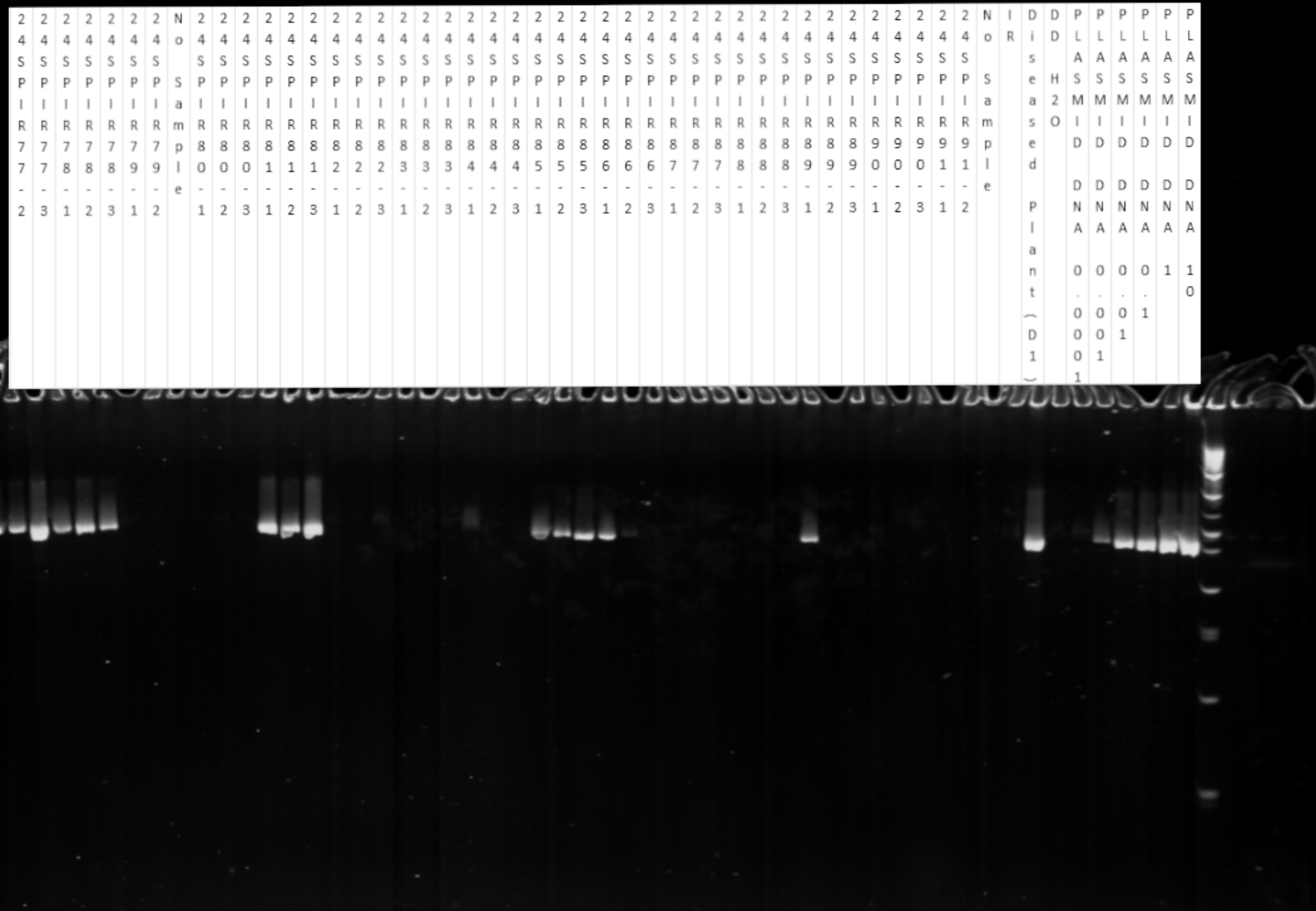
24SPIR77-2
24SPIR77-3
24SPIR78-1
24SPIR78-2
24SPIR78-3
24SPIR79-1
24SPIR79-2
No Sample
24SPIR80-1
24SPIR80-2
24SPIR80-3
24SPIR81-1
24SPIR81-2
24SPIR81-3
24SPIR82-1
24SPIR82-2
24SPIR82-3
24SPIR83-1
24SPIR83-2
24SPIR83-3
24SPIR84-1
24SPIR84-2
24SPIR84-3
24SPIR85-1
24SPIR85-2
24SPIR85-3
24SPIR86-1
24SPIR86-2
24SPIR86-3
24SPIR87-1
24SPIR87-2
24SPIR87-3
24SPIR88-1
24SPIR88-2
24SPIR88-3
24SPIR89-1
24SPIR89-2
24SPIR89-3
24SPIR90-1
24SPIR90-2
24SPIR90-3
24SPIR91-1
24SPIR91-2
No Sample
IR
Diseased Plant (D1)
DD H2O
PLASMID DNA 0.0001
PLASMID DNA 0.001
PLASMID DNA 0.01
PLASMID DNA 0.1
PLASMID DNA 1
PLASMID DNA 10

PCR program: SSR60-30 cycles

Electrophoresis: 6% acrylamide 50min

Day 3 PCR

24SPIR71-1	24SPIR71-2	24SPIR71-3	24SPIR72-1	24SPIR72-2	24SPIR72-3	24SPIR73-1	24SPIR73-2	24SPIR73-3	24SPIR74-1	24SPIR74-2	24SPIR74-3	24SPIR75-1	24SPIR75-2	24SPIR75-3	24SPIR76-1	24SPIR76-2	No Sample	24SPIR77-1	24SPIR77-2	24SPIR77-3	24SPIR78-1	24SPIR78-2	24SPIR78-3	24SPIR79-1	24SPIR79-2	No Sample	24SPIR80-1	24SPIR80-2	24SPIR80-3	24SPIR81-1	24SPIR81-2	24SPIR81-3	24SPIR82-1	24SPIR82-2	24SPIR82-3	24SPIR83-1	24SPIR83-2	24SPIR83-3	24SPIR84-1	24SPIR84-2	24SPIR84-3	24SPIR85-1

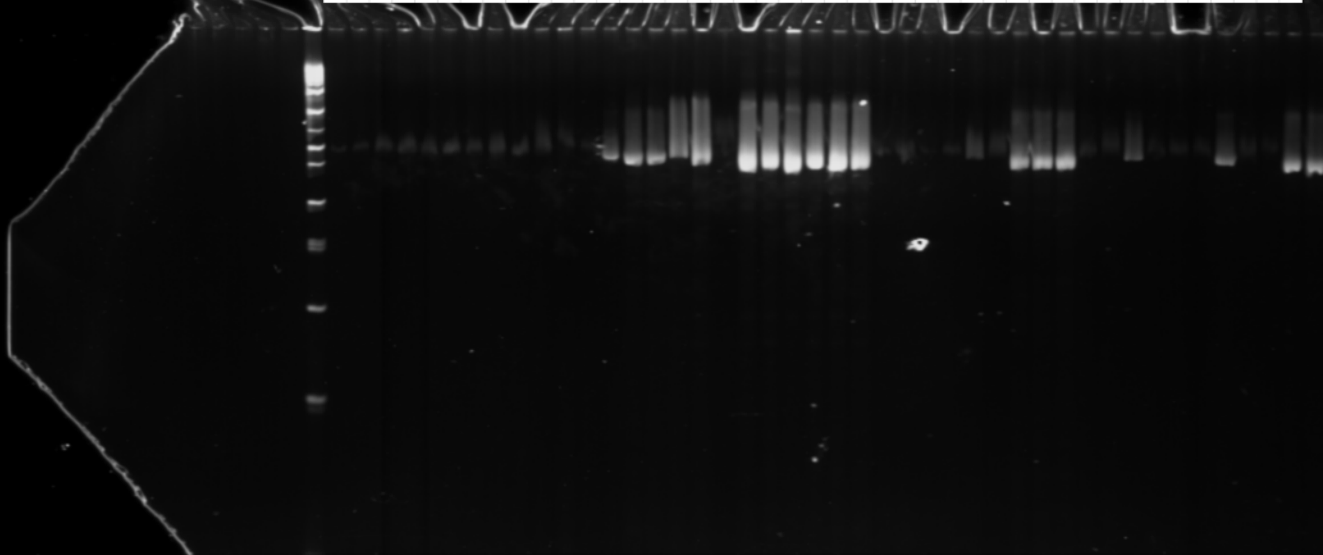

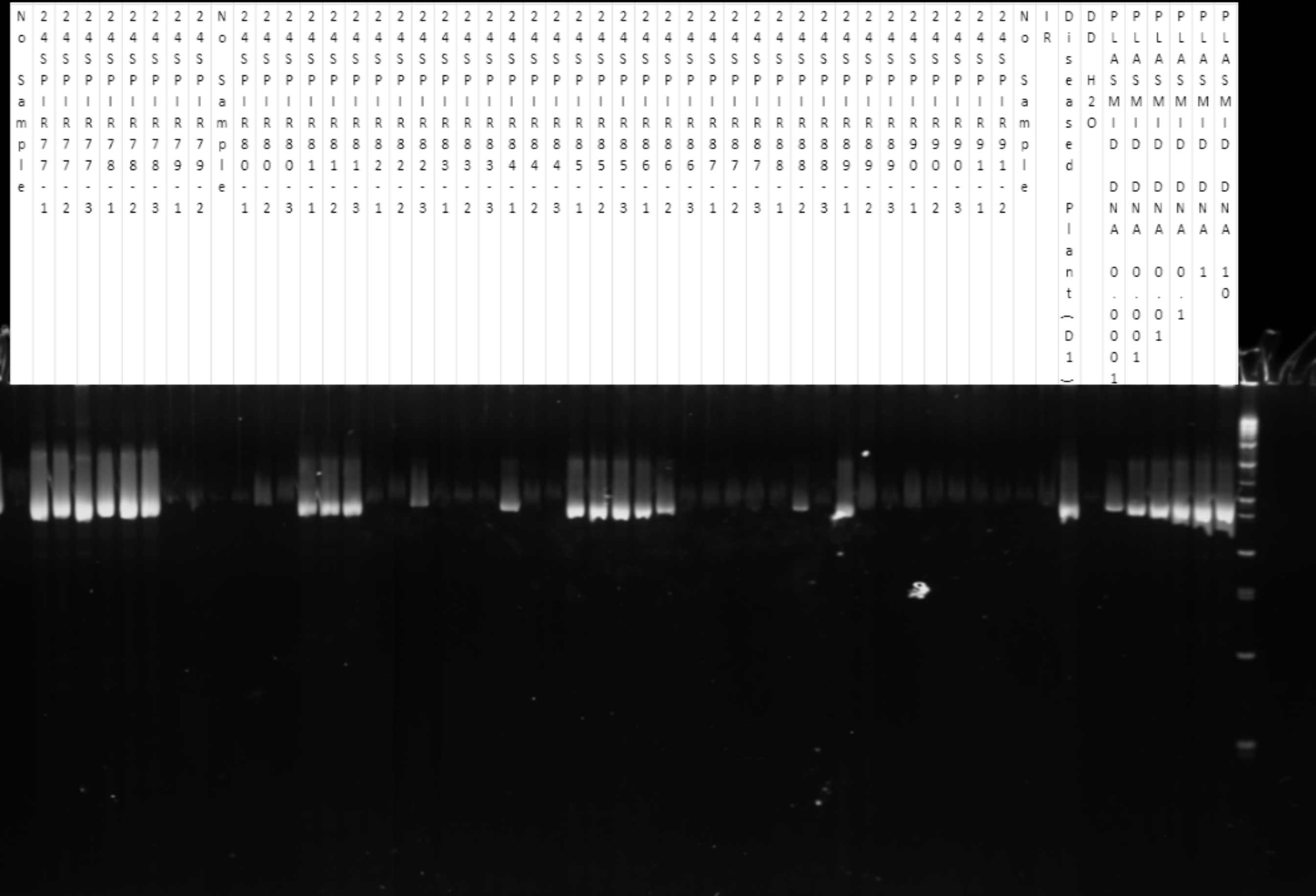
No Sample
244SPIR77-1
244SPIR77-2
244SPIR77-3
244SPIR78-1
244SPIR78-2
244SPIR78-3
244SPIR79-1
244SPIR79-2
No Sample
244SPIR80-1
244SPIR80-2
244SPIR80-3
244SPIR81-1
244SPIR81-2
244SPIR81-3
244SPIR82-1
244SPIR82-2
244SPIR82-3
244SPIR83-1
244SPIR83-2
244SPIR83-3
244SPIR84-1
244SPIR84-2
244SPIR84-3
244SPIR85-1
244SPIR85-2
244SPIR85-3
244SPIR86-1
244SPIR86-2
244SPIR86-3
244SPIR87-1
244SPIR87-2
244SPIR87-3
244SPIR88-1
244SPIR88-2
244SPIR88-3
244SPIR89-1
244SPIR89-2
244SPIR89-3
244SPIR90-1
244SPIR90-2
244SPIR90-3
244SPIR91-1
244SPIR91-2
No Sample
IR
Diseased Plant (D1)
DD H2O
PLASMID DNA 0.0001
PLASMID DNA 0.001
PLASMID DNA 0.01
PLASMID DNA 0.1
PLASMID DNA 1
PLASMID DNA 10

PCR program: SSR60-15 cycles
Electrophoresis: 6% acrylamide 50min
Day 5 PCR

24SPIR71-1	24SPIR71-2	24SPIR71-3	24SPIR72-1	24SPIR72-2	24SPIR72-3	24SPIR73-1	24SPIR73-2	24SPIR73-3	24SPIR74-1	24SPIR74-2	24SPIR74-3	24SPIR75-1	24SPIR75-2	24SPIR75-3	24SPIR76-1	24SPIR76-2	No Sample	24SPIR77-1	24SPIR77-2	24SPIR77-3	24SPIR78-1	24SPIR78-2	24SPIR78-3	24SPIR79-1	24SPIR79-2	No Sample	24SPIR80-1	24SPIR80-2	24SPIR80-3	24SPIR81-1	24SPIR81-2	24SPIR81-3	24SPIR82-1	24SPIR82-2	24SPIR82-3	24SPIR83-1	24SPIR83-2	24SPIR83-3	24SPIR84-1	24SPIR84-2	24SPIR84-3	24SPIR85-1	24SPIR85-2	24SPIR85-3	24SPIR86-1	24SPIR86-2

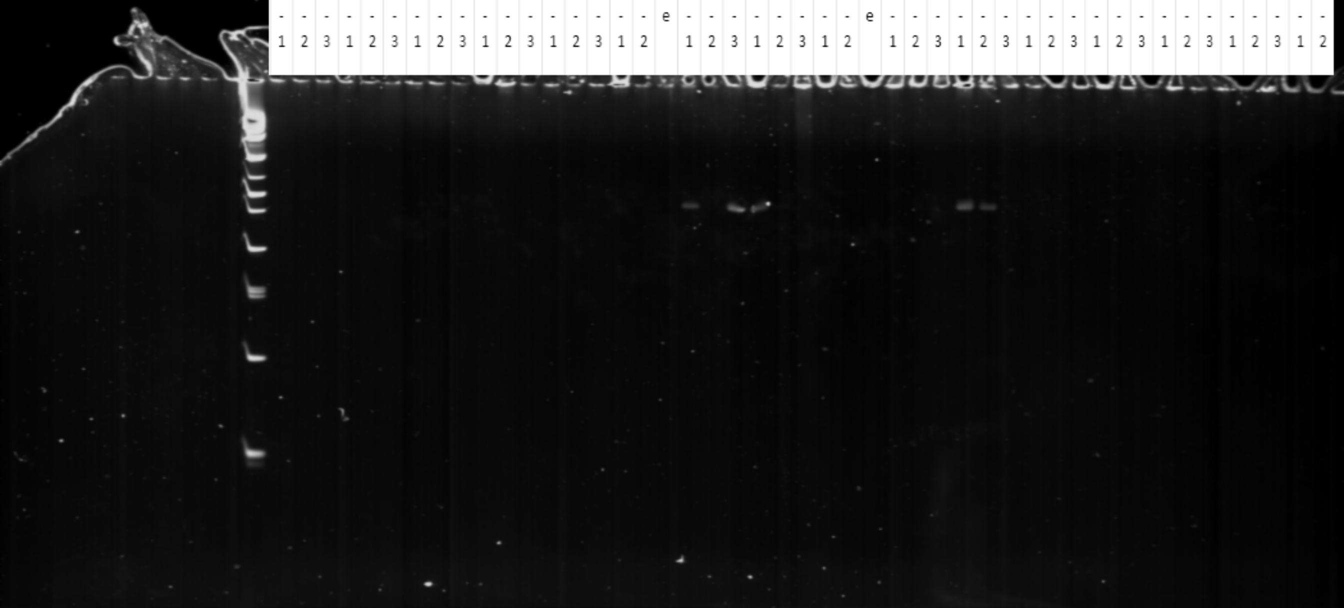

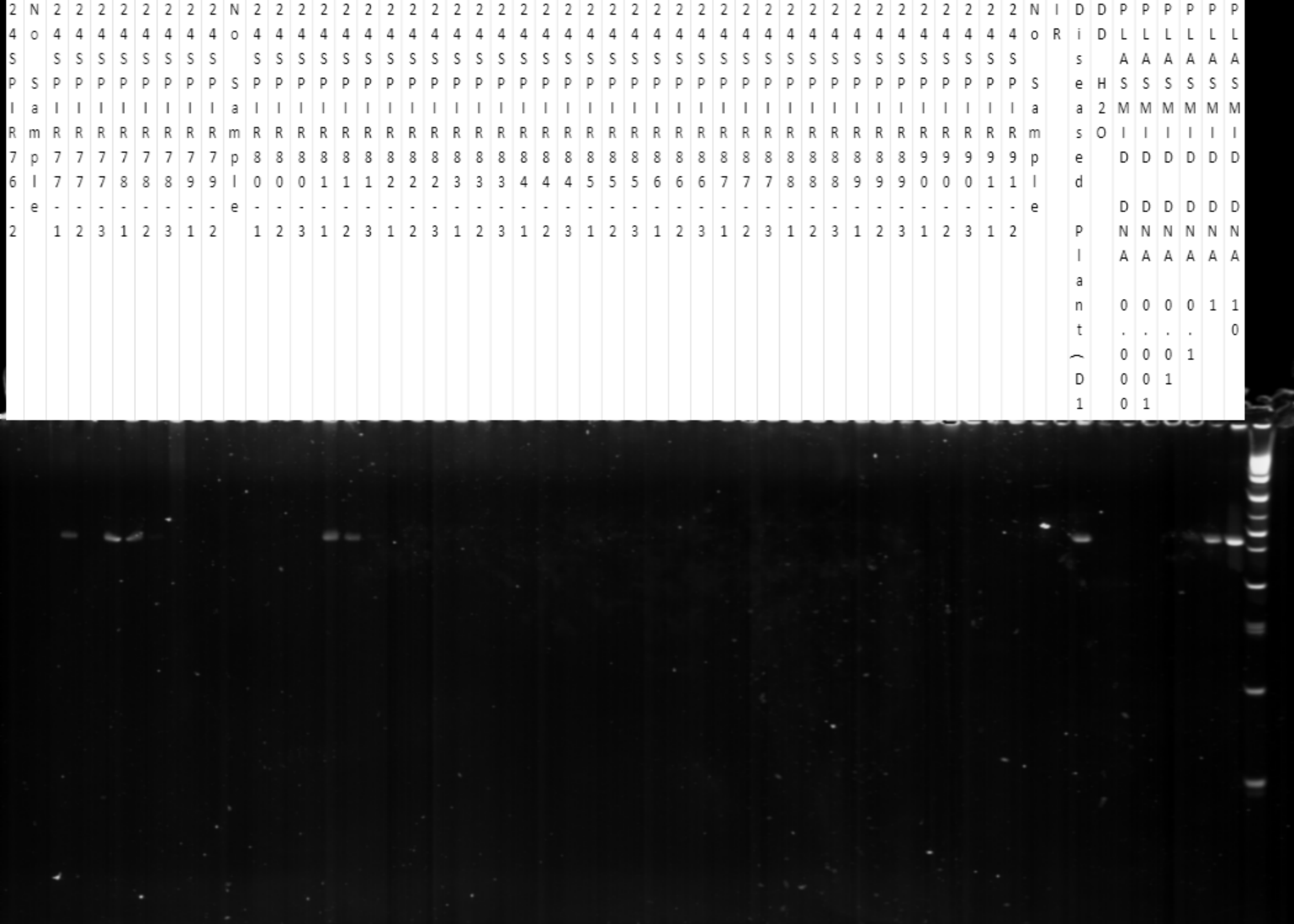
24SPIR76-2
No Sample
24SPIR77-1
24SPIR77-2
24SPIR77-3
24SPIR78-1
24SPIR78-2
24SPIR78-3
24SPIR79-1
24SPIR79-2
No Sample
24SPIR80-1
24SPIR80-2
24SPIR80-3
24SPIR81-1
24SPIR81-2
24SPIR81-3
24SPIR82-1
24SPIR82-2
24SPIR82-3
24SPIR83-1
24SPIR83-2
24SPIR83-3
24SPIR84-1
24SPIR84-2
24SPIR84-3
24SPIR85-1
24SPIR85-2
24SPIR85-3
24SPIR86-1
24SPIR86-2
24SPIR86-3
24SPIR87-1
24SPIR87-2
24SPIR87-3
24SPIR88-1
24SPIR88-2
24SPIR88-3
24SPIR89-1
24SPIR89-2
24SPIR89-3
24SPIR90-1
24SPIR90-2
24SPIR90-3
24SPIR91-1
24SPIR91-2
No Sample
IR
Diseased Plant (D1
DDH2O
PLASMID DNA 0.0001
PLASMID DNA 0.001
PLASMID DNA 0.01
PLASMID DNA 0.1
PLASMID DNA 1
PLASMID DNA 10

PCR program: SSR60-20 cycles		
Electrophoresis: 6% acrylamide 50min		
Day 5 PCR		

244SPIR71-1	244SPIR71-2	244SPIR71-3	244SPIR72-1	244SPIR72-2	244SPIR72-3	244SPIR73-1	244SPIR73-2	244SPIR73-3	244SPIR74-1	244SPIR74-2	244SPIR74-3	244SPIR75-1	244SPIR75-2	244SPIR75-3	244SPIR76-1	244SPIR76-2	No Sample	244SPIR77-1	244SPIR77-2	244SPIR77-3	244SPIR78-1

244SPIR78-2	244SPIR78-3	244SPIR79-1	244SPIR79-2	No Sample	244SPIR80-1	244SPIR80-2	244SPIR80-3	244SPIR81-1	244SPIR81-2	244SPIR81-3	244SPIR82-1	244SPIR82-2	244SPIR82-3	244SPIR83-1	244SPIR83-2	244SPIR83-3	244SPIR84-1	244SPIR84-2	244SPIR84-3

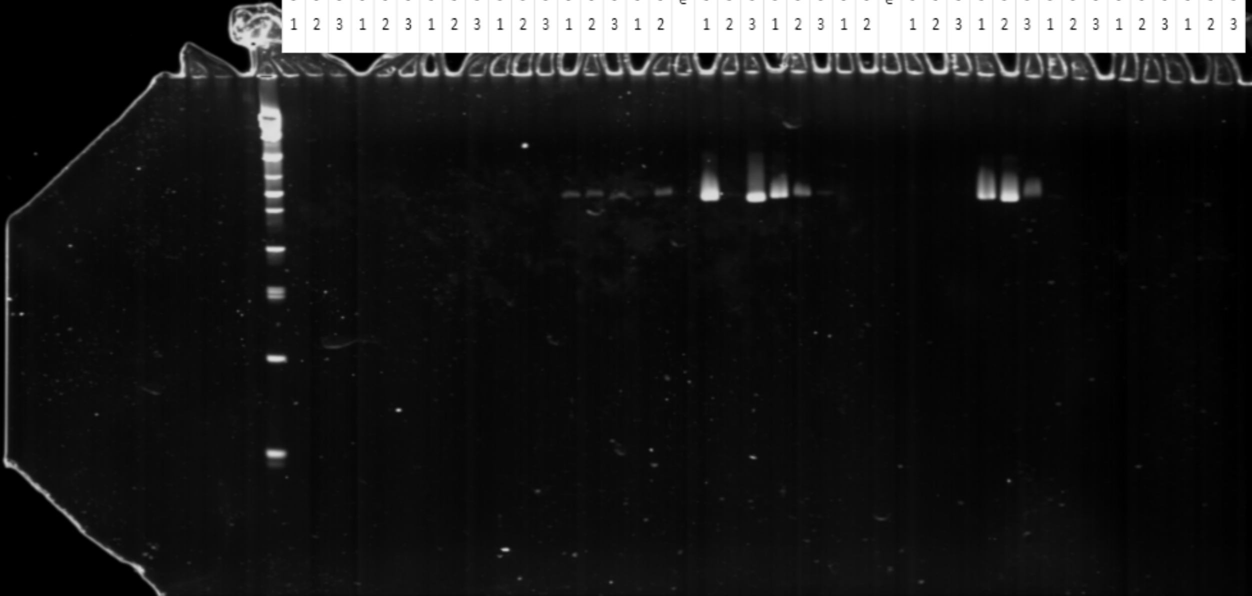

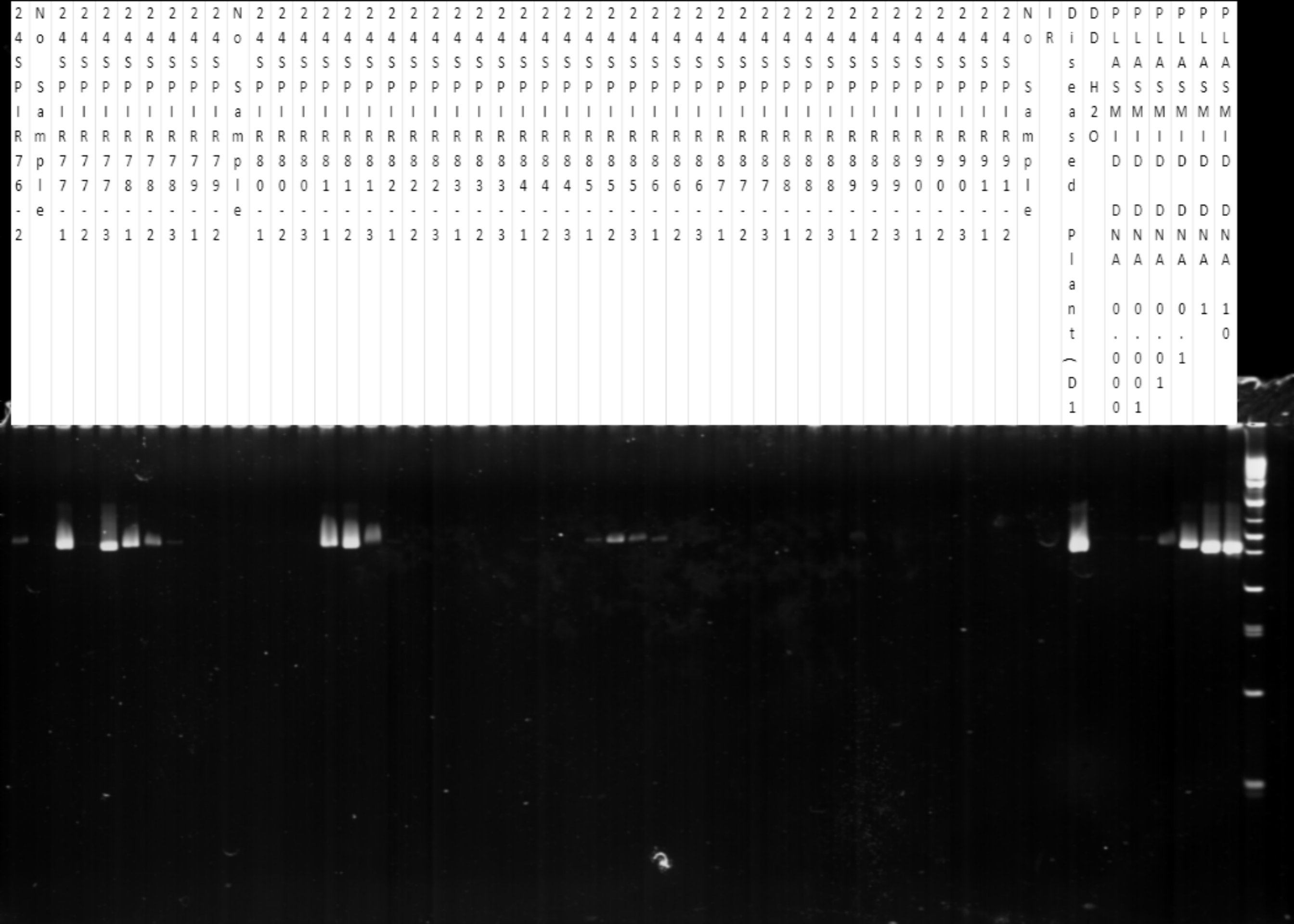

24SPIR76-2
No Sample
24SPIR77-1
24SPIR77-2
24SPIR77-3
24SPIR78-1
24SPIR78-2
24SPIR78-3
24SPIR79-1
24SPIR79-2
No Sample
24SPIR80-1
24SPIR80-2
24SPIR80-3
24SPIR81-1
24SPIR81-2
24SPIR81-3
24SPIR82-1
24SPIR82-2
24SPIR82-3
24SPIR83-1
24SPIR83-2
24SPIR83-3
24SPIR84-1
24SPIR84-2
24SPIR84-3
24SPIR85-1
24SPIR85-2
24SPIR85-3
24SPIR86-1
24SPIR86-2
24SPIR86-3
24SPIR87-1
24SPIR87-2
24SPIR87-3
24SPIR88-1
24SPIR88-2
24SPIR88-3
24SPIR89-1
24SPIR89-2
24SPIR89-3
24SPIR90-1
24SPIR90-2
24SPIR90-3
24SPIR91-1
24SPIR91-2
No Sample
IR
Diseased Plant (D1
DDH2O
PLASMID DNA 0.000
PLASMID DNA 0.001
PLASMID DNA 0.01
PLASMID DNA 0.1
PLASMID DNA 1
PLASMID DNA 10

PCR program: SSR60-25 cycles
Electrophoresis: 6% acrylamide 50min
Day 5 PCR

24SPIR71-1	24SPIR71-2	24SPIR71-3	24SPIR72-1	24SPIR72-2	24SPIR72-3	24SPIR73-1	24SPIR73-2	24SPIR73-3	24SPIR74-1	24SPIR74-2	24SPIR74-3	24SPIR75-1	24SPIR75-2	24SPIR75-3	24SPIR76-1	24SPIR76-2	No Sample	24SPIR77-1	24SPIR77-2	24SPIR77-3	24SPIR78-1	24SPIR78-2	24SPIR78-3	24SPIR79-1	24SPIR79-2	No Sample	24SPIR80-1	24SPIR80-2	24SPIR80-3	24SPIR81-1	24SPIR81-2	24SPIR81-3	24SPIR82-1	24SPIR82-2	24SPIR82-3	24SPIR83-1

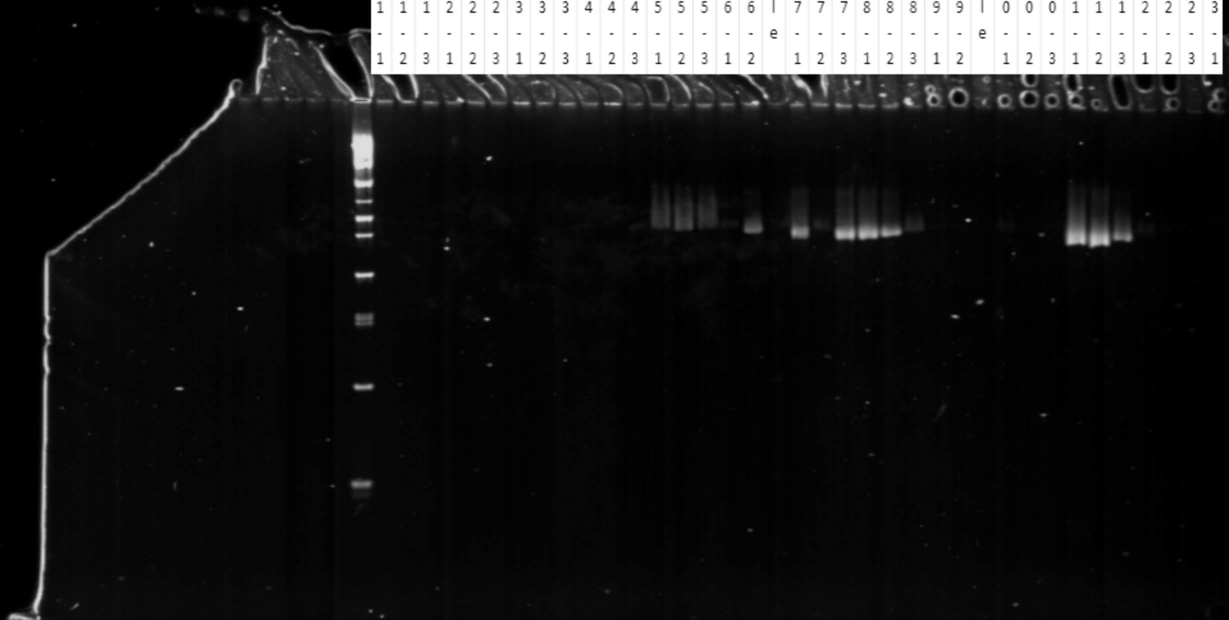

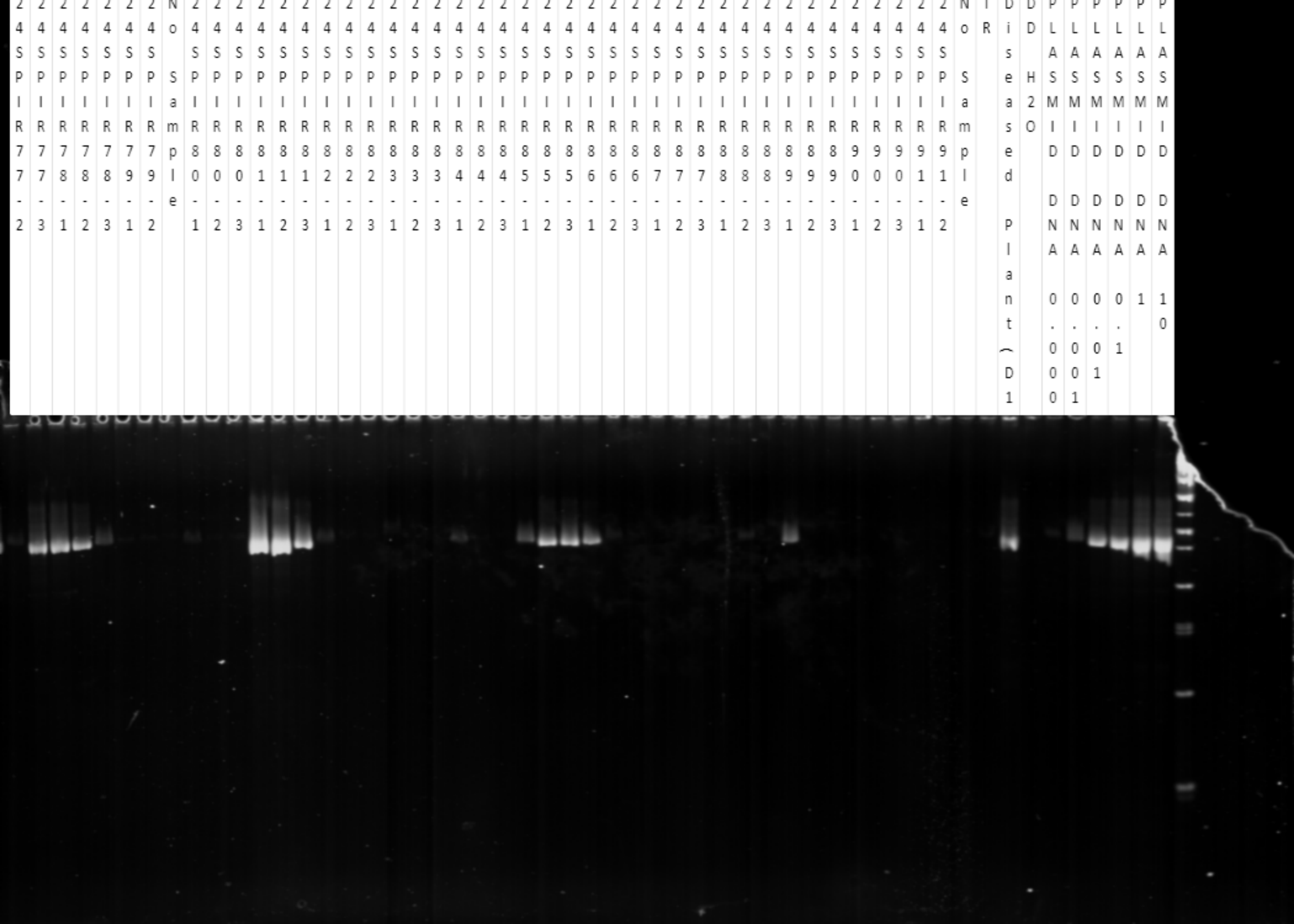

24SPIR77-2
24SPIR77-3
24SPIR78-1
24SPIR78-2
24SPIR78-3
24SPIR79-1
24SPIR79-2
No Sample
24SPIR80-1
24SPIR80-2
24SPIR80-3
24SPIR81-1
24SPIR81-2
24SPIR81-3
24SPIR82-1
24SPIR82-2
24SPIR82-3
24SPIR83-1
24SPIR83-2
24SPIR83-3
24SPIR84-1
24SPIR84-2
24SPIR84-3
24SPIR85-1
24SPIR85-2
24SPIR85-3
24SPIR86-1
24SPIR86-2
24SPIR86-3
24SPIR87-1
24SPIR87-2
24SPIR87-3
24SPIR88-1
24SPIR88-2
24SPIR88-3
24SPIR89-1
24SPIR89-2
24SPIR89-3
24SPIR90-1
24SPIR90-2
24SPIR90-3
24SPIR91-1
24SPIR91-2
No Sample
IR
Diseased Plant (D1
DDH2O
PLASMID DNA 0.0001
PLASMID DNA 0.001
PLASMID DNA 0.01
PLASMID DNA 0.1
PLASMID DNA 1
PLASMID DNA 10

PCR program: SSR60-30 cycles
Electrophoresis: 6% acrylamide 50min
Day 5 PCR

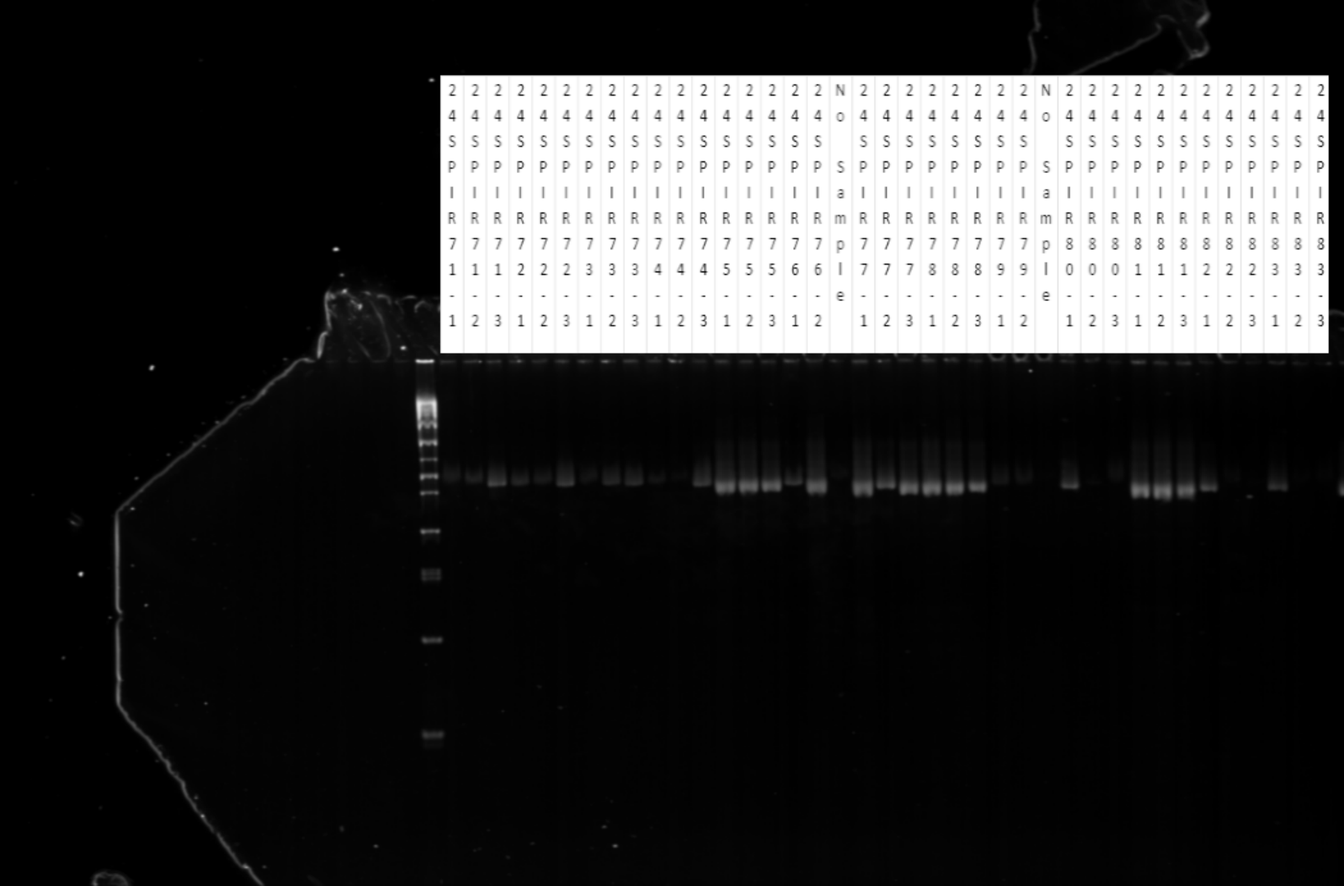

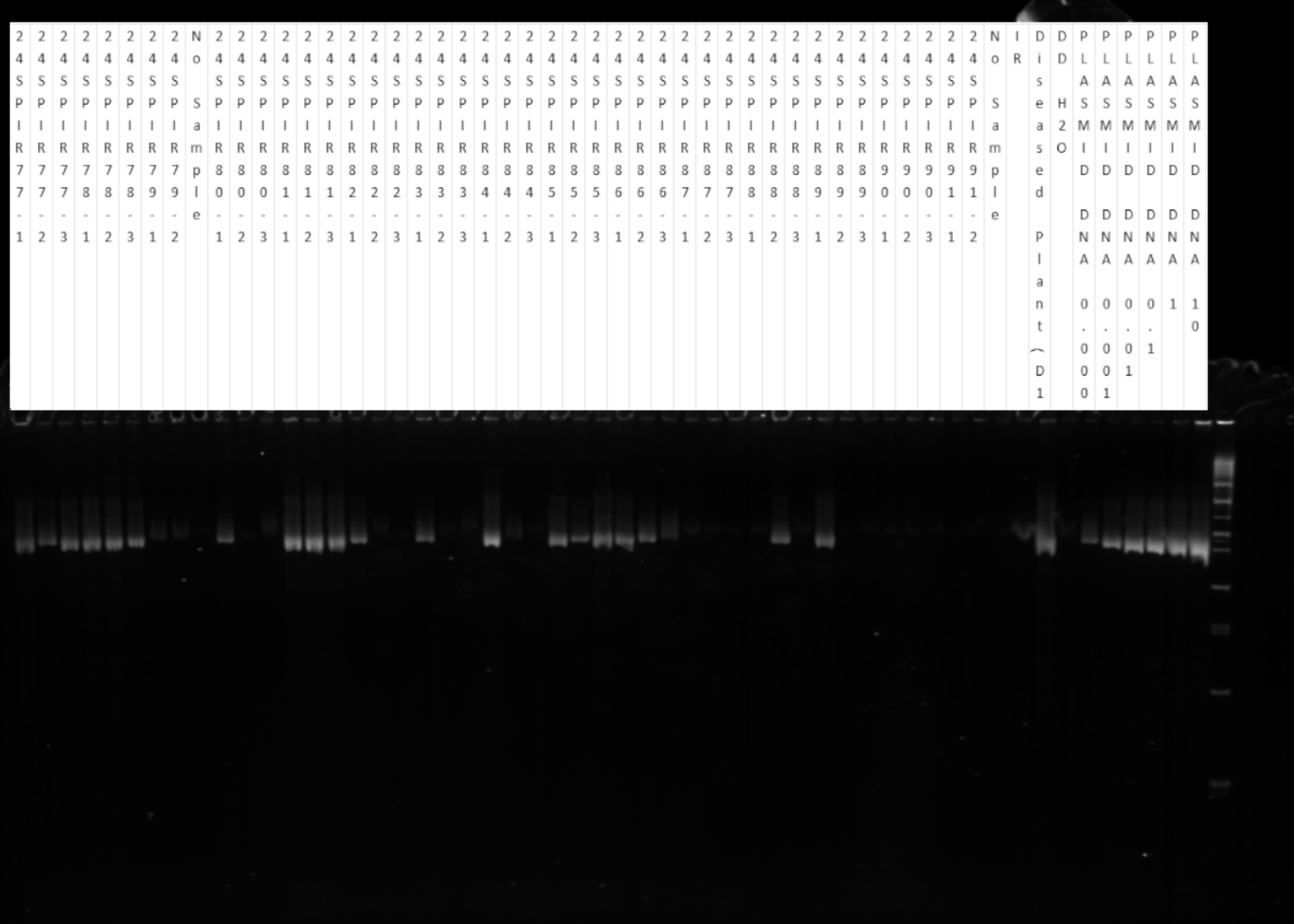
24SPIR77-1
24SPIR77-2
24SPIR77-3
24SPIR78-1
24SPIR78-2
24SPIR78-3
24SPIR79-1
24SPIR79-2
No Sample
24SPIR80-1
24SPIR80-2
24SPIR80-3
24SPIR81-1
24SPIR81-2
24SPIR81-3
24SPIR82-1
24SPIR82-2
24SPIR82-3
24SPIR83-1
24SPIR83-2
24SPIR83-3
24SPIR84-1
24SPIR84-2
24SPIR84-3
24SPIR85-1
24SPIR85-2
24SPIR85-3
24SPIR86-1
24SPIR86-2
24SPIR86-3
24SPIR87-1
24SPIR87-2
24SPIR87-3
24SPIR88-1
24SPIR88-2
24SPIR88-3
24SPIR89-1
24SPIR89-2
24SPIR89-3
24SPIR90-1
24SPIR90-2
24SPIR90-3
24SPIR91-1
24SPIR91-2
No Sample
IR
Diseased Plant(D1
DDH2O
PLASMID DNA 0.0001
PLASMID DNA 0.001
PLASMID DNA 0.01
PLASMID DNA 0.1
PLASMID DNA 1
PLASMID DNA 10

PCR program: SSR60-15 cycles							
Electrophoresis: 6% acrylamide 50min							
Day 20 PCR							

24SPIR71-1	24SPIR71-2	24SPIR71-3	24SPIR72-1	24SPIR72-2	24SPIR72-3	24SPIR73-1	24SPIR73-2	24SPIR73-3	24SPIR74-1	24SPIR74-2	24SPIR74-3	24SPIR75-1	24SPIR75-2	24SPIR75-3	24SPIR76-1	24SPIR76-2	No Sample	24SPIR77-1	24SPIR77-2	24SPIR77-3	24SPIR78-1	24SPIR78-2	24SPIR78-3	24SPIR79-1	24SPIR79-2	No Sample	24SPIR80-1	24SPIR80-2	24SPIR80-3	24SPIR81-1	24SPIR81-2	24SPIR81-3	24SPIR82-1	24SPIR82-2	24SPIR82-3	24SPIR83-1	24SPIR83-2	24SPIR83-3	24SPIR84-1	24SPIR84-2	24SPIR84-3	24SPIR85-1	24SPIR85-2	24SPIR85-3	24SPIR86-1	24SPIR86-2	24SPIR86-3	24SPIR87-1	24SPIR87-2	24SPIR87-3	24SPIR88-1

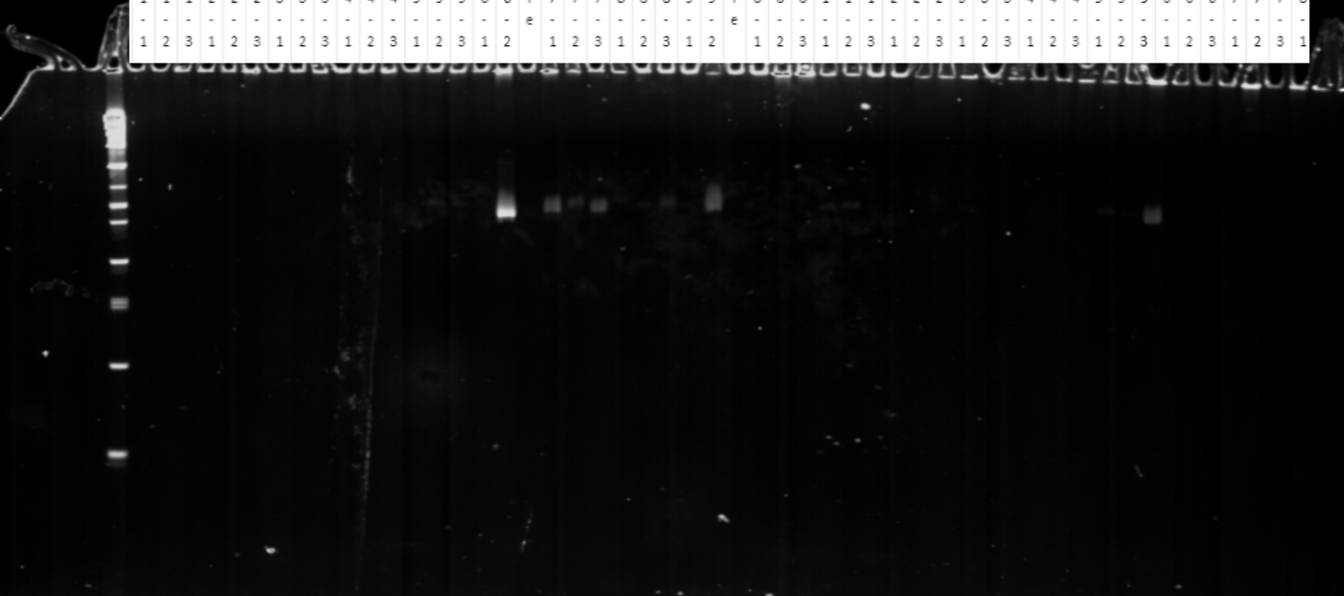

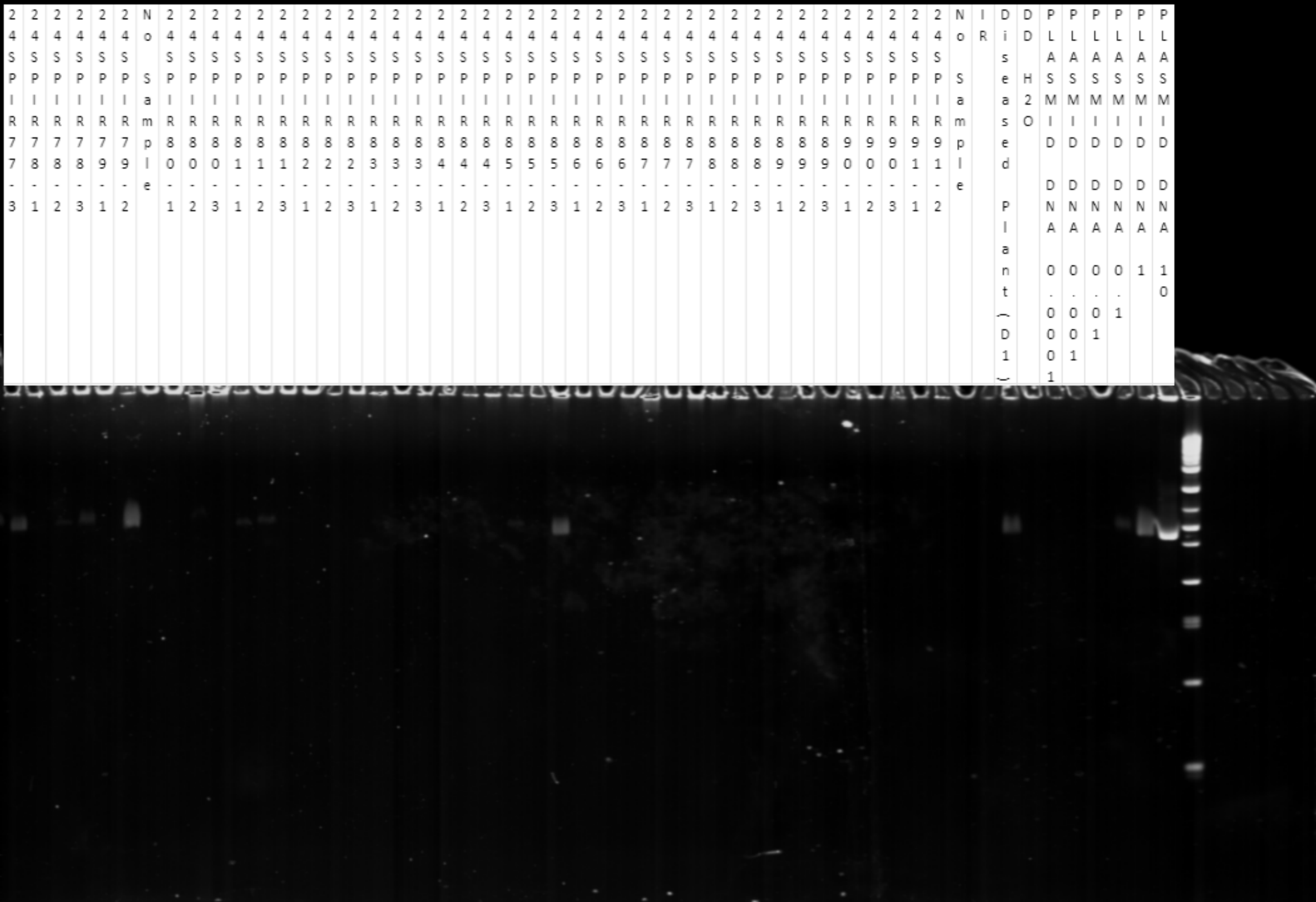
24SPIR77-3
24SPIR78-1
24SPIR78-2
24SPIR78-3
24SPIR79-1
24SPIR79-2
No Sample
24SPIR80-1
24SPIR80-2
24SPIR80-3
24SPIR81-1
24SPIR81-2
24SPIR81-3
24SPIR82-1
24SPIR82-2
24SPIR82-3
24SPIR83-1
24SPIR83-2
24SPIR83-3
24SPIR84-1
24SPIR84-2
24SPIR84-3
24SPIR85-1
24SPIR85-2
24SPIR85-3
24SPIR86-1
24SPIR86-2
24SPIR86-3
24SPIR87-1
24SPIR87-2
24SPIR87-3
24SPIR88-1
24SPIR88-2
24SPIR88-3
24SPIR89-1
24SPIR89-2
24SPIR89-3
24SPIR90-1
24SPIR90-2
24SPIR90-3
24SPIR91-1
24SPIR91-2
No Sample
IR
Diseased Plant (D1)
DDH2O
PLASMID DNA 0.0001
PLASMID DNA 0.001
PLASMID DNA 0.01
PLASMID DNA 0.1
PLASMID DNA 1
PLASMID DNA 10

PCR program: SSR60-20 cycles

Electrophoresis: 6% acrylamide 50min

Day 20 PCR

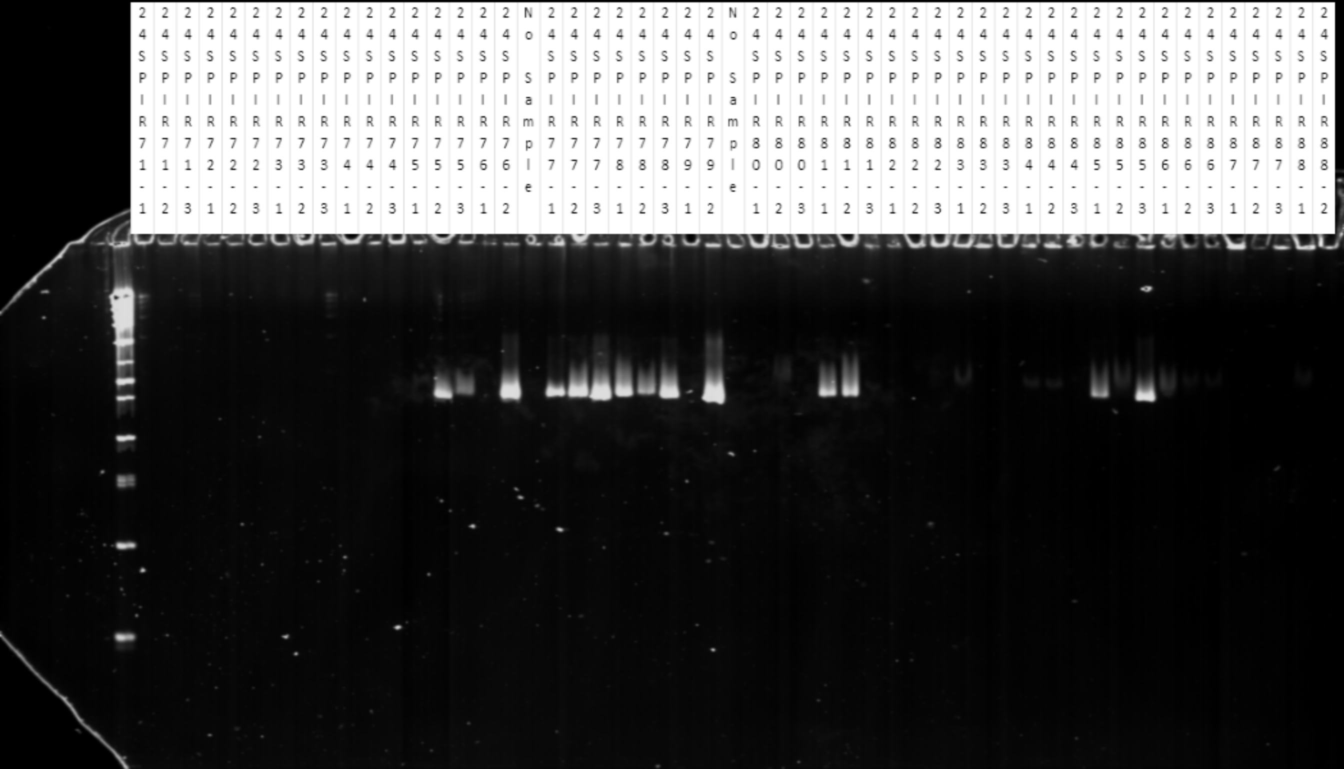

24SPIR80-2	24SPIR80-3	24SPIR81-1	24SPIR81-2	24SPIR81-3	24SPIR82-1	24SPIR82-2	24SPIR82-3	24SPIR83-1	24SPIR83-2	24SPIR83-3	24SPIR84-1	24SPIR84-2	24SPIR84-3	24SPIR85-1	24SPIR85-2	24SPIR85-3	24SPIR86-1	24SPIR86-2	24SPIR86-3	24SPIR87-1	24SPIR87-2	24SPIR87-3	24SPIR88-1	24SPIR88-2	24SPIR88-3	24SPIR89-1	24SPIR89-2	24SPIR89-3	24SPIR90-1	24SPIR90-2	24SPIR90-3	24SPIR91-1	24SPIR91-2	No Sample	IR	Diseased Plant (D1)	DD H2O	PLASMID DNA 0.0001	PLASMID DNA 0.001	PLASMID DNA 0.01	PLASMID DNA 0.1	PLASMID DNA 1	PLASMID DNA 10

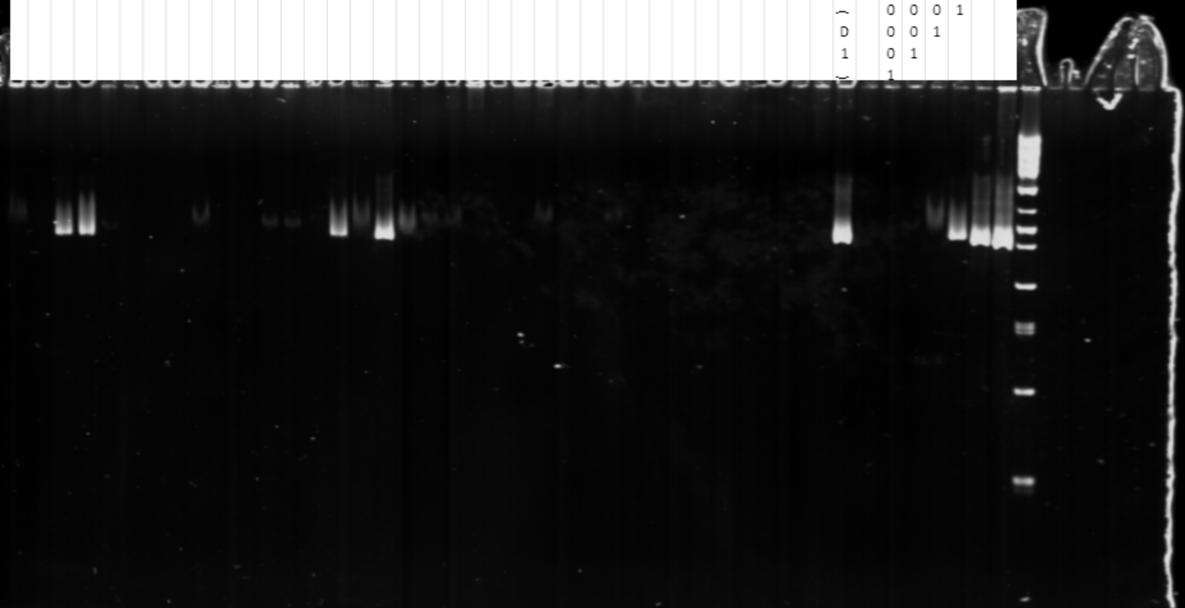

PCR program: SSR60-25 cycles
Electrophoresis: 6% acrylamide 50min
Day 20 PCR

24SSPIR71-1	24SSPIR71-2	24SSPIR71-3	24SSPIR72-1	24SSPIR72-2	24SSPIR72-3	24SSPIR73-1	24SSPIR73-2	24SSPIR73-3	24SSPIR74-1	24SSPIR74-2	24SSPIR74-3	24SSPIR75-1	24SSPIR75-2	24SSPIR75-3	24SSPIR76-1	24SSPIR76-2	No Sample	24SSPIR77-1	24SSPIR77-2	24SSPIR77-3	24SSPIR78-1	24SSPIR78-2	24SSPIR78-3	24SSPIR79-1	24SSPIR79-2	No Sample	24SSPIR80-1	24SSPIR80-2	24SSPIR80-3	24SSPIR81-1	24SSPIR81-2	24SSPIR81-3	24SSPIR82-1	24SSPIR82-2	24SSPIR82-3	24SSPIR83-1	24SSPIR83-2	24SSPIR83-3	24SSPIR84-1	24SSPIR84-2	24SSPIR84-3	24SSPIR85-1	24SSPIR85-2	24SSPIR85-3	24SSPIR86-1	24SSPIR86-2	24SSPIR86-3	24SSPIR87-1	24SSPIR87-2

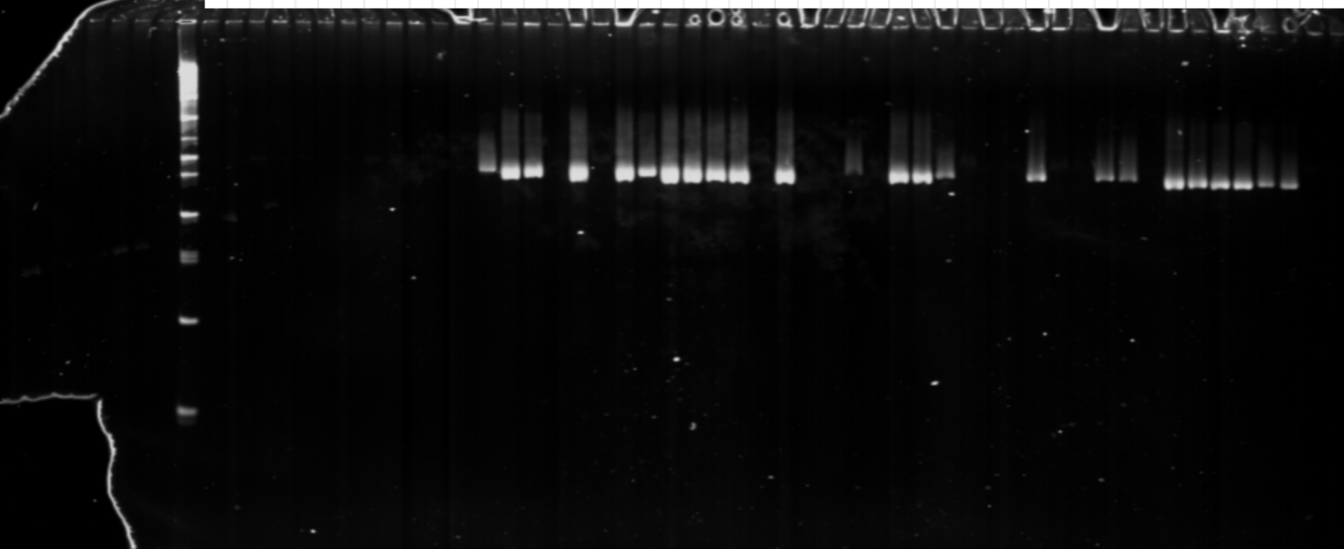

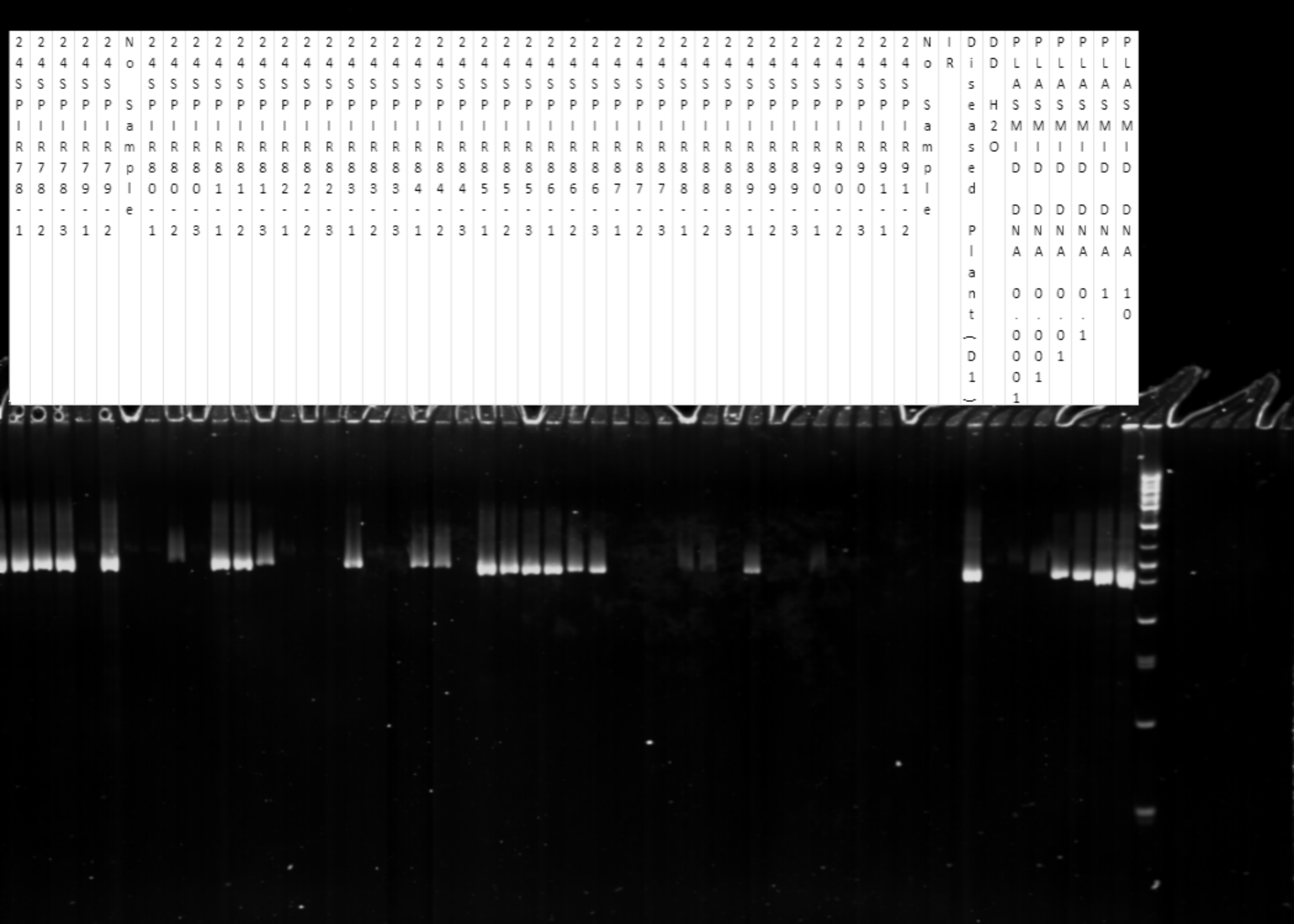
24SPIR78-1
24SPIR78-2
24SPIR78-3
24SPIR79-1
24SPIR79-2
No Sample
24SPIR80-1
24SPIR80-2
24SPIR80-3
24SPIR81-1
24SPIR81-2
24SPIR81-3
24SPIR82-1
24SPIR82-2
24SPIR82-3
24SPIR83-1
24SPIR83-2
24SPIR83-3
24SPIR84-1
24SPIR84-2
24SPIR84-3
24SPIR85-1
24SPIR85-2
24SPIR85-3
24SPIR86-1
24SPIR86-2
24SPIR86-3
24SPIR87-1
24SPIR87-2
24SPIR87-3
24SPIR88-1
24SPIR88-2
24SPIR88-3
24SPIR89-1
24SPIR89-2
24SPIR89-3
24SPIR90-1
24SPIR90-2
24SPIR90-3
24SPIR91-1
24SPIR91-2
No Sample
IR
Diseased Plant (D1)
DDH2O
PLASMID DNA 0.0001
PLASMID DNA 0.001
PLASMID DNA 0.01
PLASMID DNA 0.1
PLASMID DNA 1
PLASMID DNA 10

PCR program: SSR60-30 cycles
Electrophoresis: 6% acrylamide 50min
Day 20 PCR

24SPIR71-1	24SPIR71-2	24SPIR71-3	24SPIR72-1	24SPIR72-2	24SPIR72-3	24SPIR73-1	24SPIR73-2	24SPIR73-3	24SPIR74-1	24SPIR74-2	24SPIR74-3	24SPIR75-1	24SPIR75-2	24SPIR75-3	24SPIR76-1	24SPIR76-2	No Sample	24SPIR77-1	24SPIR77-2	24SPIR77-3	24SPIR78-1	24SPIR78-2	24SPIR78-3	24SPIR79-1	24SPIR79-2	No Sample	24SPIR80-1	24SPIR80-2	24SPIR80-3	24SPIR81-1	24SPIR81-2	24SPIR81-3	24SPIR82-1	24SPIR82-2	24SPIR82-3	24SPIR83-1	24SPIR83-2	24SPIR83-3	24SPIR84-1	24SPIR84-2	24SPIR84-3	24SPIR85-1	24SPIR85-2	24SPIR85-3	24SPIR86-1	24SPIR86-2	24SPIR86-3	24SPIR87-1	24SPIR87-2	24SPIR87-3	24SPIR8[illegible]-1

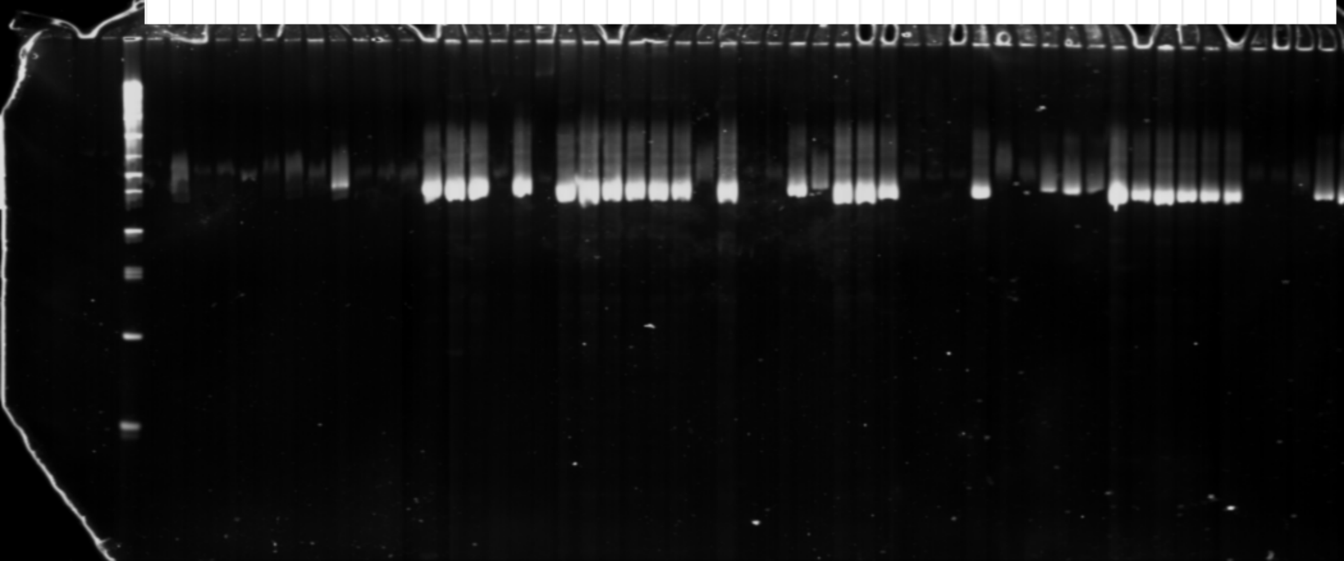

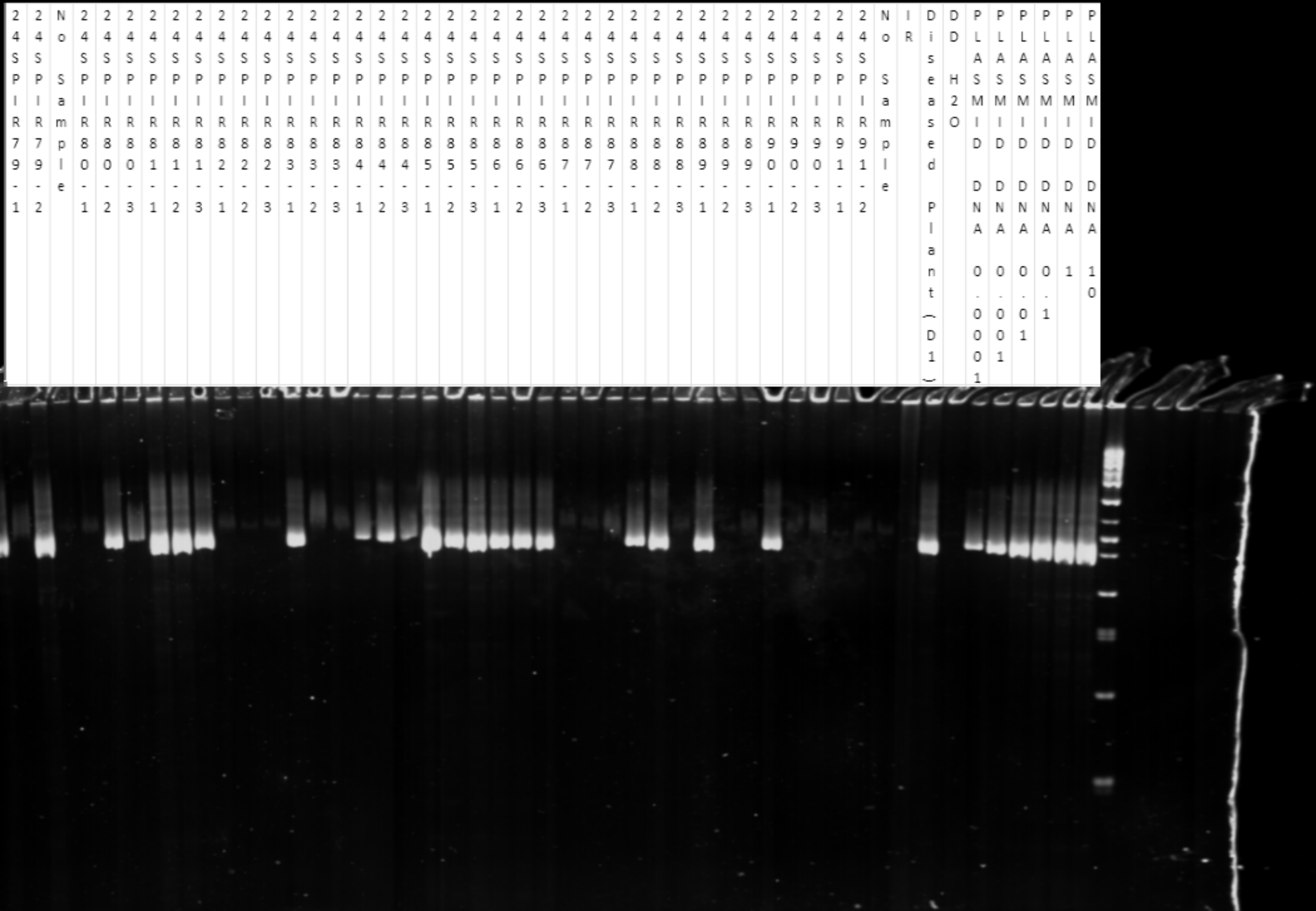
24SPIR79-1
24SPIR79-2
No Sample
24SPIR80-1
24SPIR80-2
24SPIR80-3
24SPIR81-1
24SPIR81-2
24SPIR81-3
24SPIR82-1
24SPIR82-2
24SPIR82-3
24SPIR83-1
24SPIR83-2
24SPIR83-3
24SPIR84-1
24SPIR84-2
24SPIR84-3
24SPIR85-1
24SPIR85-2
24SPIR85-3
24SPIR86-1
24SPIR86-2
24SPIR86-3
24SPIR87-1
24SPIR87-2
24SPIR87-3
24SPIR88-1
24SPIR88-2
24SPIR88-3
24SPIR89-1
24SPIR89-2
24SPIR89-3
24SPIR90-1
24SPIR90-2
24SPIR90-3
24SPIR91-1
24SPIR91-2
No Sample
IR
Diseased Plant (D1)
DD H2O
PLASMID DNA 0.0001
PLASMID DNA 0.001
PLASMID DNA 0.01
PLASMID DNA 0.1
PLASMID DNA 1
PLASMID DNA 10
