# Supplementary material for: Enhanced Tomato Yellow Leaf Curl Thailand Virus Suppression Through Multi-Disease and Insect-Resistant Tomato Lines Combining Virus and Vector Resistance
Source: Insects. 2025 Jul 15;16(7):721. doi: 10.3390/insects16070721 (PMC12295848; doi:10.3390/insects16070721)
Supplement: Supplementary file 1 [file insects-16-00721-s001.zip › Text S1. Protocol for DNA extraction from tomato leaf samples using a 96-well plate.pdf]

DNA extraction for Tomato (using 96 well-plate)

(According to Tanksley Lab, modified by AVRDC)

1. Add few 3 mm glass bead (5~7) into 96 well-plate. Collect 2 leaf tissues from a 3-4 leaf stage into the 96 well-plate.
2. Prepare fresh Microprep Buffer, keep at 65℃ waterbath.
3. Add 275 ul fresh Microprep Buffer I (Sodiumdisulfite + Solution 1) to each well, shake the plate with Mini Beadbeater for 30-60 sec till no tissue observed. Spin down for 20 sec.
4. Add 375 ul (125*3) Microprep Buffer II+III (lysis buffer+ sarcosyl stock) (need to be kept warm) to each well, shake to mix well. Spin down for 20 sec.
5. Incubate for 60-90 min at 65℃.
6. Centrifuge plates at 6,000 rpm for 10~20 min.
7. Pipette off aqueous phase (200-220 ul) into new 96 well-plate.
8. Add 2/3 to 1 times the volume of cold isopropanol (200 ul) to each well. Invert tubes gentle until the DNA precipitates (mostly invert 20~30 times).
   (DNA will begin to become visible as milky/clear cloud of fibbers in the solution.)
9. Centrifuge tubes at 6,000 rpm for 10~20 min and discard supernatant.
10. Add 0.6 ml 70% ethanol.

    (You can stop in this step, keep you sample in -20℃.)
11. Centrifuge tubes at 6,000 rpm for 5~10 min and discard ethanol.
    (If color is green or brown, wash with 70% ethanol again)
12. Dry the pellet by Freeze Dryer for 1 hour, or left them in the clean place.
13. Resuspend the pellet in 200-300 μl of distilled water at 65℃ for 10 min or at 4℃ for overnight.

    (can treat with RNase, incubate at 37℃ for 1~2 hours, then heat inactivate at 80℃ for 15 min)
14. Store the DNA at 4°C for up to 1 week or -20 °C for longer storage.

Stock buffer solution:

**1 M Tris**	**→ can use 1M Tris-HCl (pH 8.0)**
**0.5 M EDTA (pH 7.5)**	**→ can use 0.5 M EDTA (pH 8.0)**

**Extraction Buffer (stock at 4℃):**		**(to make 500 ml)**
0.35 M	Sorbitol	= 31.9 g Sorbitol
0.1 m	Tris	= 50 ml 1 M Tris
5 mM	EDTA	= 5 ml 0.5 M EDTA (pH 7.5)
--> Adjust to pH 7.5 with HCl		→ Fill up to 500 ml with MilliQ H_2O

**Nuclei Lysis Buffer (stock at RT):**		**(to make 500 ml)**
0.2 M	Tris	= 100 ml 1 M Tris
0.05 M	EDTA	= 50 ml 0.5 M EDTA (pH 7.5)
2 M	NaCl	= 200 ml 5 M NaCl
2 %	CTAB	= 10 g CTAB
		→ Fill up to 500 ml with MilliQ H_2O

5% (w/v) Sarcosyl(Sarkosyl) stock (stock at RT):

(N-LAUROYL SARCOSINE)

12.5 g / 250 ml

Microprep Buffer:

	03 ml	30 ml	60 ml	90 ml	120 ml	130 ml	150 ml
Sodiumdisulfite (= sodium metabisulfite)	0.01 g	0.1 g	0.2 g	0.3 g	0.4 g	0.44 g	0.5 g
PVP (poly(vinylpolypyrolidone))	0.06 g	0.6 g	1.2 g	1.8 g	2.4 g	2.64 g	3 g
extraction buffer	1.25 ml	12.5 ml	25 ml	37.5 ml	50 ml	55 ml	62.5 ml
→ mix well in 65℃ waterbath							
lysis buffer	1.25 ml	12.5 ml	25 ml	37.5 ml	50 ml	55 ml	62.5 ml
sarcosyl stock	0.5 ml	5 ml	10 ml	15 ml	20 ml	22 ml	25 ml

p/s:

1. This buffer may settle into two layers on standing. Heat to 65℃ and shake immediately before adding to the extraction tubes.
2. This buffer also can use directly without settle into two layers, use the magnet to stir when adding to the tubes.
3. The PVP (poly(vinylpolypyrolidone)) can be use by half.

TE Buffer

	100 ml
1 M Tris-Cl (pH7.5 or 8.0)	1 ml
0.5 M EDTA (pH 7.5 or 8.0)	0.2 ml

→ Fill up to 100 ml with MilliQ H_2O
