# Supplementary material for: Enhanced Tomato Yellow Leaf Curl Thailand Virus Suppression Through Multi-Disease and Insect-Resistant Tomato Lines Combining Virus and Vector Resistance
Source: Insects. 2025 Jul 15;16(7):721. doi: 10.3390/insects16070721 (PMC12295848; doi:10.3390/insects16070721)
Supplement: Supplementary file 1 [file insects-16-00721-s001.zip › Text S2. Protocol for acylsugar quantification in plant samples.pdf]

Mutschler Lab
Cornell University

Standard PGO based Acylsugar Assay (Last updated 1/2010)

Supplies needed, and their preparation.

A. Sample Collection

1. **Scintillation vials for collecting samples.**
Options:

- Glass vials (VWR 56941-513 500/case capped, $131.51) have the advantage of being clear which aids visualization when rinsing the samples later in the assay. They also usually come with the racks needed to handle large sample sizes.
- Plastic **vials** (VWR 66021-704 1000/case loose with separate caps $143.80), have a slightly narrower mouth than the glass vials but work well, and are about half the price.
- **WE PREFER**: Large mouth plastic vials from LPS (L212200 500/case loose with separate caps $52.89). These are less expensive than the VWR plastic vials and their large mouth and semi-transparency makes them easy to use. You can also buy them racked (L212201 100/rack 500/case $60.80) and re-use the racks for subsequent assays. These need to be ordered about a month in advance since they are frequently back ordered.
- It is advantageous to order the vials NOT capped. Otherwise, someone will have to uncap all of the vials before going to the field. If there are 2000 or so vials, that would take a long time.

2. **Forceps.** Ones with a moderately fine point capable of breaking off leaflet samples but not so sharp that the leaf samples are torn. We have used VWR 25716-002 5/pack $8.38.

3. **Gloves.** Gloves are recommended to prevent hands from getting soiled from the tomato plants.

4. **An exact listing of plants in the field to be assayed** to use in labeling vials. Precision in this list will prevent preparation of vials for plants that have died, or preparation of too few vials. Precision here also minimizes the likelihood of human error in sample collection.

5. **Labels for vials:** We generally use return address labels because they are large enough for field numbers-sample number information, small enough to only cover part of the vial wall, and easy to print out using most word processing packages.

6. **Lightweight stools and folding tables** help as well, if many samples are being collected in a field situation,

B. Acylsugar assay

1. **Re-pipettor** makes it easier to accurately dispense the same volume of methanol each time to many vials.
2. **Strip tubes in racks.** Corning Co-Star 4412 (Fisher 07-200-321 10 racks/case $54.60.
3. **Strip tubes (if already have racks to reuse).** We use Corning Co-Star 4408 (Fisher 07-200-318 120 strips of 8 tubes/case $34.67).
4. **Strip tube caps.** Corning Co-Star 4418 (Fisher 07-200-323 120 strips of 8 caps/case $17.34).
5. **Pipette tips.** 1ml (1000ul) and 200ul tips are needed.
6. **Microtiter plates.** We use VWR 82050-760 100/case $44.72.
7. **Microtiter plate covers.** We use Falcon 3913 (Fisher 08-772-10 50/box $39)
8. **Sticky PCR plate covers.** We use LPS SecureSeal (LPS T329002 100/box $60.73)
9. **Reagents as indicated in protocol.**
10. **Mulitchannel pipettors** save a lot of time and aid in accuracy when performing the assay.

Preparation before collecting samples

Do all of the preparation work well in advance. It takes longer than one would guess to collect when the number of samples is large. Good preparation saves time in the field, and reduces the likelihood of error.

1. Make labels for vials There is a separate protocol sheet for this step. We generally collect two sample vials per plant (A and B). For some experiments we use four samples per plant (A though D). On the label indicate :

row (or genotype) - ID number within row – sample letter (e.g. 017197-7 A).

2. Place a label on each vial near the neck and rack the vials. We arrange vials back to front, then left to right in numerical order. It can help to keep subgroups in separate trays. Always bring extra vials with blank labels, in case one has to hand label an additional vial.

Collecting and drying leaflet samples

For each vial, pluck 3 leaflets from a leaf at the third or fourth node down from the stem apex of the appropriate plant using forceps. Choose intact leaflets without holes, leaf damage, honeydew, etc. since free sugars from inside or on the surface may bias the assay.

- The goal is to collect leaflets that have neither lost sample or been contaminated by sample from neighboring plants. Do not use leaves facing or drooping into the alley; if the plants are very large, leaves are likely to have been brushed against by staff walking around. Similarly, do not collect from branches interlaced in neighboring plants.
- The lateral leaflets of the compound leaf of tomato leaflets tend to be longer/narrower than the terminal leaflet on a leaf. Narrower is better, since it is easier to put the leaflet into the vial properly.
- The aggregate leaflet area should be as similar as possible in all of the samples collected. We generally collect leaflets the size of three or four quarters, preferably four quarters.
- A sample that is too small will distort the results of the assay. A sample that is too large will crowd the vial, slow sample drying, interfere with sample washing and distort the results. Try to be consistent in sample collection.
- When inserting the leaflet into the vial, do so in such a way that the leaflet does not touch the outside of the vial, only the inside of the vial so that sample is not lost.
- Do not touch the sample with your fingers or you will remove acylsugar.

After leaves are all collected, dry the samples in the racked vials in a drying oven about 2 or 3 days. We have generally used forage driers. Set the temperature at 80 to 85 degree F, not higher. (28 c) Air movement helps. We want to dry down, not bake, the samples.

Preparation of Samples:

1) Prepare wash sample maps and assay plate maps. These show which samples will be stored in which positions with the strip tubes, which are in a 96 well plate format.

    - **We generally use the first column in the 96 well assay plate for the standard curve samples.** To represent the standards, leave the first row blank for both the sample map and the assay map in order to maintain the consistency of both maps and reduce error. This is particularly important when thousands of samples are being tested.

    Insert strip tubes into holders, and label each rack to correspond to one of the racks in the sample maps.

2) Calibrate the repipetor before use with a 10ml glass graduated cylinder,

3) Rinse dried leaf samples and collect rinsate sample

- Add desired volume of methanol to a dried leaf sample using repipetor, (rechecking calibration periodically between samples).
- The volume of methanol can be 2 to 6ml (and the value used is recorded, so it can be accounted for in later calculations). If acylsugar levels are expected to be high (greenhouse grown plants of fixed lines) use a higher volume. If acylsugar levels are expected to be low (plants in cool field conditions, segregating populations) use a lower volume. We generally use 3ml.
- Immediately after adding methanol, cap the vial, invert a couple of times and hand-swirl for a second or two. Vortexing is NOT recommended because it will fragment the dried tissue, and the resultant tissue fragments will contaminate the sample with free sugars and cause significant error.
- Remove 1ml of solution to appropriate strip tube, taking care that no tissue is transferred, and the liquid is only deposited in the appropriate tube. If the solution is green, **STOP washing**, and determine and correct the problem. Samples may be over-washed or fragments of leaves were transferred to the strip tubes.

4) When a whole row of strip tubes has been filled, seal it with lids to minimize evaporation and the chance for cross contamination.

5) Store racks of samples in -20C freezer until assay performed.

6) Alternatively, if time/manpower permits, reaction plates (see below) can be prepared simultaneously as each 96 tube rack is completed and before the tubes are sealed. This saves the time/effort of taking the lids off the strip tubes later. The plate is used for the assay; the tubes are capped and stored in the freezer, in case the assay must be repeated.

7) After all the dried leaf samples in the vials have been washed and sampled, place the tray in the hood to dry overnight. Keep trays for future leaf weights.

- Weigh leaf tissue after all of the remaining alcohol has evaporated, and the samples are fully dried.
- Some top loading scales can be programmed and linked to a PC so that the weights are recorded automatically. Doing this reduces error and saves time.
- Alternatively, prepare a spreadsheet listing samples, manually record leaf weights and type data into spreadsheet
- For breeding purposes, in segregating populations we only measure and record weights of samples that have acylsugars, as seen by their absorbance values. This greatly reduces the number of samples to weigh.

Assay Day 1:

1) Prepare assay maps in advance.

2) Row 1 of the 96 well format will hold 100ul of appropriate standard curve.
   - This would be a glucose or sucrose standard curve, depending on whether the samples will be tested minus or plus invertase, respectively.
   - Our current acylsugar lines only produce acylsucrose, so we only run plus invertase assay.
   - Newer populations are segregating for acylglucose production. When both acylsucrose and acylglucose can be produced, we run the assay twice, once with invertase and once without invertase, to be able to detect and calculate levels of both acylsucroses and acylglucoses, as described below.

3) It's a good idea to include a few wells with methanol blanks in some of the plates to serve as measurements of the blank plate plus reagents.
   - This allows a check for contamination of reagents.
   - These samples are not included in the calculation of the regression line for the standard curve. We have found that the regression lines for the assays do not intersect the origin: the assay is not linear at the lowest extremes. More accurate linear standard curves are obtained when one does NOT use a methanol blank within the standard curve.

4) Transfer 100ul of each sample to be assayed to the appropriate wells of rows 2 through 12 of microtiter plate using a multichannel pipette. If doing multiple plates at once, cover each plate with a microtiter plate cover until all the plates are done. This reduces evaporation of the sample.

5) Add 100ul 6M ammonium hydroxide to each well of the plate in a fume hood.

6) Seal with a sticky PCR plate cover and incubate for 2 hours in the fume hood.

7) Remove covers and allow samples to dry down in the fume hood (*ca.* two days).

- If the samples are still wet then incubate in a 60C oven until completely dried.

- It is CRITICAL that you DO NOT proceed with Day 2 of the assay until all samples are completely dry. Wet wells will give spurious results.

Assay Day 2

1) Add 200ul PGO reagents.

2) Incubate at least 2 hours (3 is better) at room temperature. Gentle rotation on an orbital shaker can reduce time needed for uniform color development. Whatever incubation time is used, you must use the same time for all plates.

3) Read plate using spectrophotometer set at 490nm (red). Any bubbles in the wells will affect the reading, so remove them by spinning briefly in a centrifuge.

4) Download results into excel.

Calculations

1. Transfer data (absorbance and leaflet dry weight) into appropriate columns, on the same spreadsheet.

2. Calculate experiment wide standard curve using the absorbance data from all of the plates. This generally results in an equation for a straight line, and an r^2 value of 0.98 or higher. If there is more scatter, and the r^2 value is lower, one could use the reiterative process to correct the standard curve. But we have not needed to do so.

3. Embed the new regression equation into the appropriate cell in the spread sheet.

4. Make sure that the cell with the volume of methanol for rinsing and of volume of sample used in the assay are correct in their column.

5. Then do the Acylsugar calculations

Please note: For testing plants that can produce both or either/or, acylsucrose and acylglucose, the situation is more complex, and one tests as follows

- Perform assay both with invertase and without invertase for all samples.
- The plus invertase assay uses sucrose standard curve, the minus invertase assay uses a glucose standard curve.
- Prepare enough stock PGO reagent for both plates and add peroxidase and glucose oxidase. Then split the reagent into two equal portions and add invertase to only one portion. The plus invertase reagent is used on the total acylsugar plate with sucrose standards while the minus invertase reagent is added to the acylglucose plate with glucose standards.
- Use the linear equation derived from the glucose standard curve, the glucose absorbances, and the tissue weights to calculate production of acylglucoses in mM/gm dried tissue.
- For each sample, subtract the sample absorbance minus invertase from the sample absorbance plus invertase. This difference is the absorbance attributable from acylsucroses. Use this value, the linear equation derived from the sucrose standard curve, and the tissue weights to calculate production of acylsucroses mM/gm dried tissue.
- Add the acylsucrose and acylglucose estimates to determine total acylsugars mM/gm dried tissue.
- Use total acylsugar production and acylglucose production levels to calculate % acylglucose production.

SUCROSE OR GLUCOSE STANDARDS

1. The stock sucrose and glucose solutions are

Glucose FW: 180.20 50ml of 0.00133M glucose stock

Sucrose FW: 342.3 50ml of 0.00133M sucrose stock

To make these stock solutions, follow the 3 step protocols

Step 1 for Glucose:
20mg glucose/ml ddH_2O

Step 2 for Glucose (0.0055M):
1ml of step 1 solution into 19ml of 100% methanol to get 20ml step 2 solution. This results in 1mg glucose/ml methanol (0.0055M) step 2 soln.

Step 3 for Glucose. To make final stock solution (0.00133M):
12 ml of step 2 solution into 38ml of 100% Methanol to get 50ml of stock soln.

Step 1 for Sucrose:
37.98mg sucrose/ml ddH_2O

Step 2 for Sucrose (0.0055M):
1ml of step 1 solution into 19ml of 100% methanol to get 20ml step 2 solution. This results in 1.899mg sucrose/ml methanol (0.0055M) step 2 soln.

Step 3 for Sucrose. To make final stock solution (0.00133M):
12 ml of step 2 solution into 38ml of 100% Methanol to get 50ml of stock soln.

Once the stock solutions are made, cap the bottles VERY TIGHLTLY, seal with parafilm, and store in the freezer.

2. Using stocks to create the standard curve solutions

Accuracy in preparation of the standard solutions is critical to accuracy in the assay.

Make enough standard curve solution so that the same standard curve solutions are used for an entire experiments assay

Once the Standard solutions are made, cap the bottles VERY TIGHLTLY, seal with parafilm, and store in the freezer.

You may want to load and keep the standard curves in strip tubes using a 96 well format (just before doing assay). This way the standard curve samples are easier to load in the microtiter plate.

We have used different standard curves, depending on the expected levels of acylsugars in the assay. We want the bulk of the samples to have absorbances in the mid range of the values of the standard curve and minimize samples with absorbances beyond the range of the standard curve. The standard curve that we have generally used follows:

Standard curve generally used.

Std ID#	Mix to Make 10 ml of Standards		Final Glucose (mg/ml)	Final Sucrose (mg/ml)	Final Glucose or Sucrose (Mol/L)
	Methanol (ml)	Stock Solution			
1	8.75	1.25	0.03	0.057	0.00017
2	7.50	2.50	0.06	0.114	0.00033
3	6.25	3.75	0.09	0.171	0.00050
4	5.00	5.00	0.12	0.228	0.00067
5	3.75	6.25	0.15	0.285	0.00083
6	2.50	7.50	0.18	0.342	0.00100
7	1.25	8.75	0.21	0.399	0.00117
8 (stock)	0.00	10.00	0.24	0.456	0.00133

3. **Diluted standard curves.** When acylsugars levels of are expected to be somewhat lower, a standard curve with a correspondingly low range is desired.

Diluted standards were used in summers with very cool field conditions for extended period of time.

One could create a diluted standard by diluting each of the normal solutions similarly. (A 50:50 mix of a standard solution and methanol, for example.)

One can also create a diluted standard using the stock solution. The following diluted standards were used in 2007.

Diluted standard curve

ID#	Mix to Make 10 ml of Standards		Final Glucose (mg/ml)	Final Sucrose (mg/ml)	Final Glucose or Sucrose (Mol/L)
	Methanol (ml)	stock solution (ml)			
1D	9.75	0.25	0.006	0.011394	0.000033
2D	8.5	1.5	0.036	0.068364	0.000200
3D	7.25	2.75	0.066	0.125334	0.000366
4D	6	4	0.096	0.182304	0.000532
5D	4.75	5.25	0.126	0.239275	0.000698
6D	3.5	6.25	0.156	0.296244	0.000865
7D	2.25	7.75	0.186	0.353214	0.001031
8D	1	9	0.216	0.410184	0.001197

OTHER REAGENTS

1. 6 M ammonium hydroxide

To make 500ml **6 M ammonium hydroxide**

Combine 362.62 ml of 29%NH_4OH into137.3 ml ddH_2O

2. PGO reagent (Peroxidase/Glucose Oxidase)

A) PGO stock without enzymes

1) Dissolve in 200 ml ddH_2O:
   3.4 g KH_2PO_4
   0.75 g para-hydroxybenzoic acid
   0.025 g 4-aminoantipyrene
   0.25 g Bovine serum albumin(BSA)---Sigma :A8022
   1.25 ml of 2% Sodium Azide stock
2) Adjust pH to 7.0 with 2N NaOH
3) Bring volume up to 250 ml
4) Store at 4 C

B) PGO with enzymes (store at 4 C), ready – to-- use

Add enzymes in 250 ml PGO stock:

250 units (2.21mg) peroxidase----Sigma: P-8125
500 units (86.2ul) glucose oxidase----Sigma: G-0543

4 mg invertase ----Sigma: I-4504 (for total acylsugar assay only)
