# Supplementary figures and images for: Enhanced Tomato Yellow Leaf Curl Thailand Virus Suppression Through Multi-Disease and Insect-Resistant Tomato Lines Combining Virus and Vector Resistance
Source: Insects. 2025 Jul 15;16(7):721. doi: 10.3390/insects16070721 (PMC12295848; doi:10.3390/insects16070721)

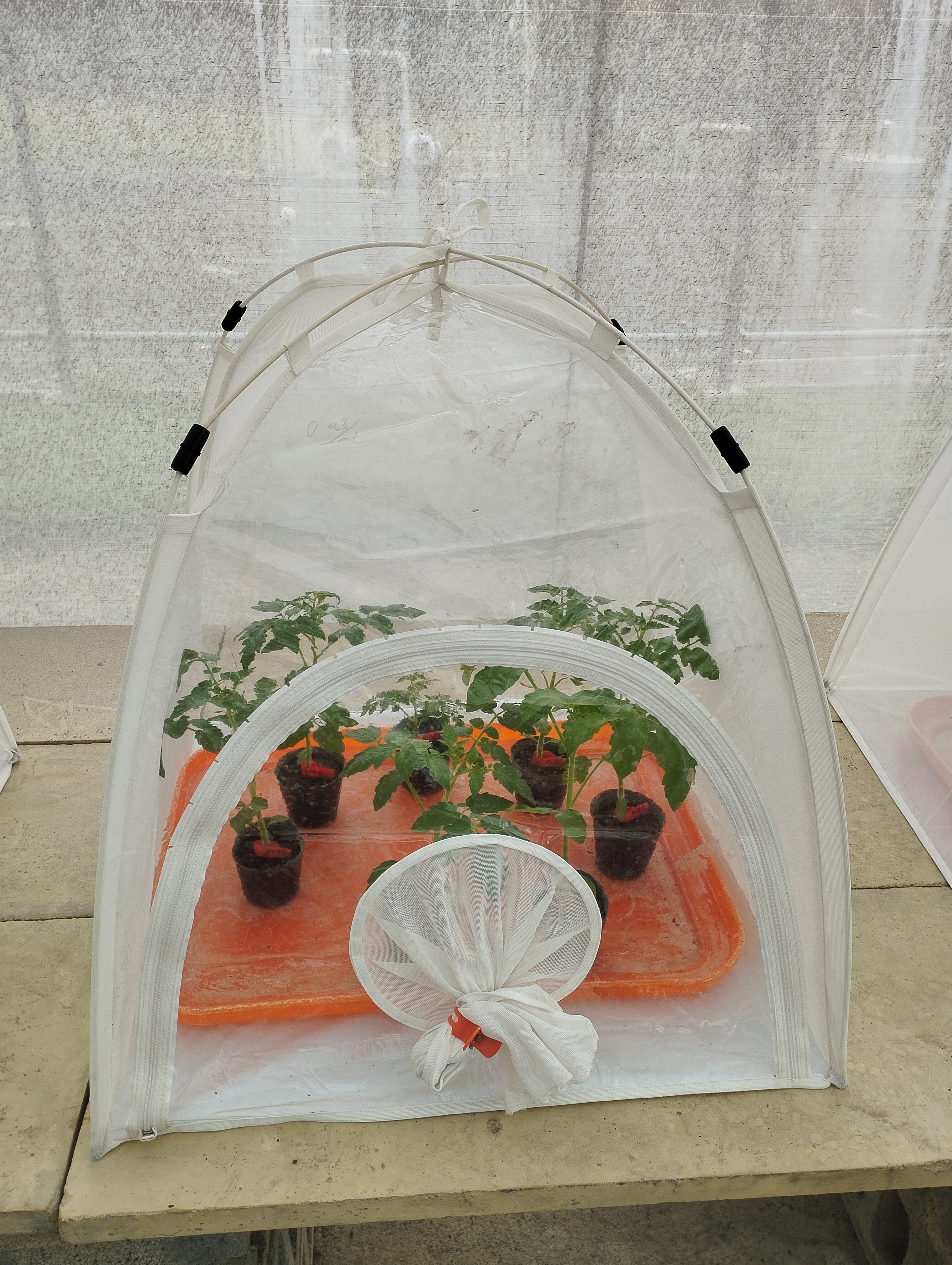

Supplement: Supplementary file 1 [file insects-16-00721-s001.zip › Figure S1. Photo of choice assay setup 1.jpg]

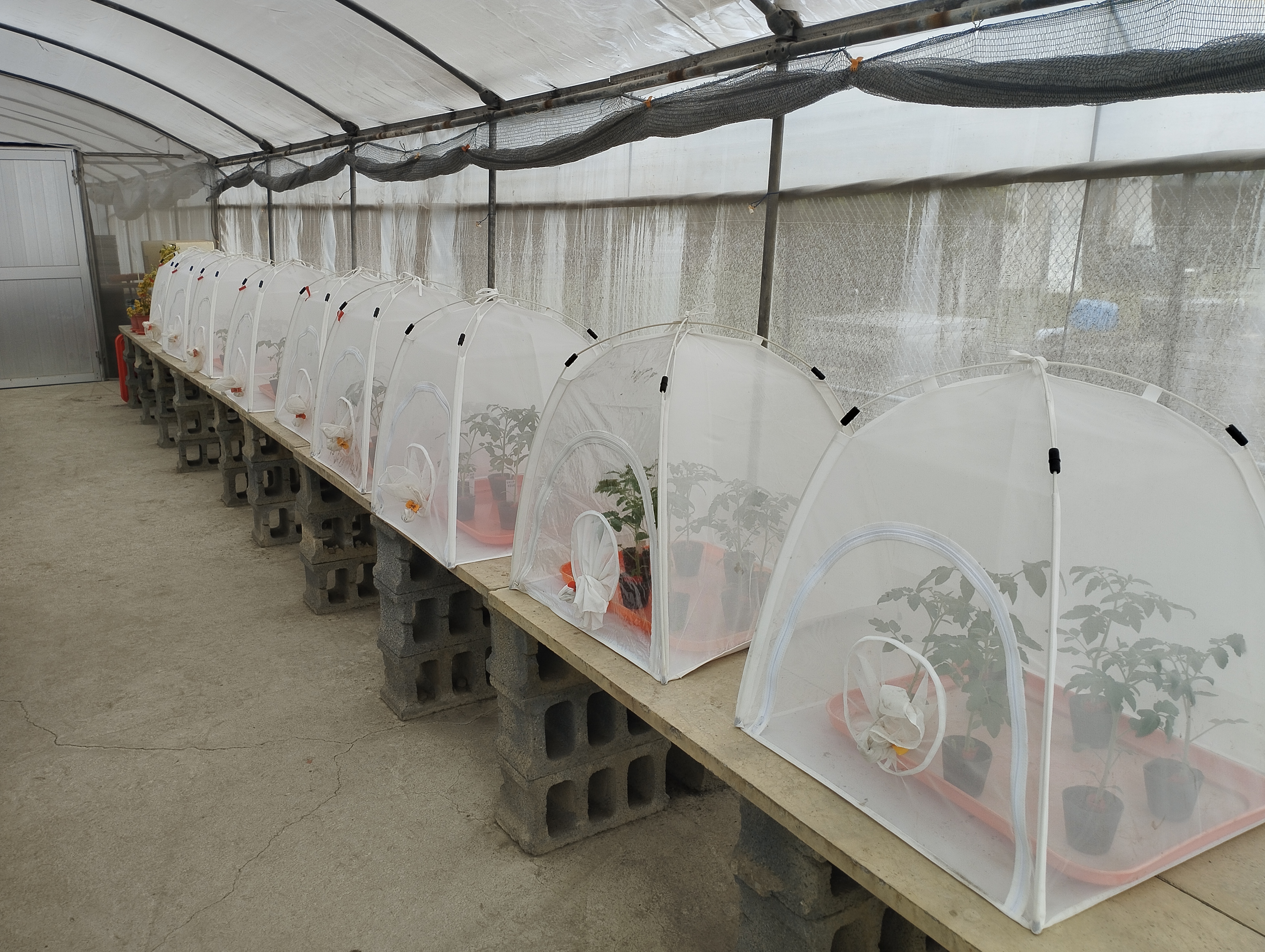

Supplement: Supplementary file 1 [file insects-16-00721-s001.zip › Figure S2. Photo of choice assay setup 2.jpg]
